# Impact of Ramadan fasting on serum levels of major endocrinology hormonal and biochemical parameters in healthy non-athlete adults: A systematic review and meta-analyses

**DOI:** 10.1371/journal.pone.0299695

**Published:** 2024-05-23

**Authors:** Mohammad Poursalehian, Shahrzad Mohseni, Zhaleh Shadman, Mohammadreza Mohajeri-Tehrani, Rasha Atlasi, Mohsen Khoshniat Nikoo, Mostafa Qorbani, Bagher Larijani

**Affiliations:** 1 Endocrinology and Metabolism Research Center, Endocrinology and Metabolism Clinical Sciences Institute, Tehran University of Medical Sciences, Tehran, Iran; 2 Students’ Scientific Research Center (SSRC), Tehran University of Medical Sciences, Tehran, Iran; 3 Elderly Health Research Center, Endocrinology and Metabolism Population Sciences Institute, Tehran University of Medical Sciences, Tehran, Iran; 4 Diabetes Research Center, Endocrinology and Metabolism Clinical Sciences Institute, Tehran University of Medical Sciences, Tehran, Iran; 5 Non-Communicable Diseases Research Center, Alborz University of Medical Sciences, Karaj, Iran; Qatar University, College of Health Sciences, QATAR

## Abstract

**Background:**

Ramadan Intermittent Fasting (RIF) has the potential to alter hormonal levels in the body. This study investigates the impact of RIF on hormonal levels among healthy individuals during Ramadan.

**Methods:**

A systematic review and meta-analysis of previously published studies were conducted, focusing on healthy non-athlete adults. The intervention examined was Ramadan Intermittent Fasting, and the primary outcomes assessed were changes in endocrine hormonal and biochemical parameters. The pooled effect measure was expressed as odds ratio (OR) and 95% confidence interval (CI) using the random-effects model.

**Results:**

A total of 35 original articles were retrieved, with a combined sample size of 1,107 participants eligible for the meta-analysis. No significant relationship was found between pre- and post-Ramadan hormonal levels of T3, T4, TSH, FT3, FT4, Testosterone, LH, FSH, Prolactin, PTH, Calcium, and Phosphorus (P-value<0.05). However, a substantial decrease in morning cortisol levels was observed across the studies (P-value: 0.08, Hedges’ g = -2.14, 95% CI: -4.54, 0.27).

**Conclusions:**

Ramadan Intermittent Fasting results in minimal hormonal changes and is a safe practice for healthy individuals. The fasting regimen appears to disrupt the circadian rhythm, leading to a decrease in morning cortisol levels.

## Introduction

Ramadan, the ninth month of the Islamic lunar calendar, holds significant religious importance for the global Muslim community. One of the Five Pillars of Islam, Ramadan, mandates a month-long period of fasting from dawn to dusk for healthy adult Muslims [1, 2]. This period of intermittent fasting, which extends roughly between 11 to 18 hours per day, is a unique metabolic state that has sparked considerable scientific interest over the past few decades [3].

Given the shift in eating patterns, energy consumption, and sleep cycles, Ramadan fasting can influence various physiological and biological processes, potentially affecting the body’s hormonal and biochemical landscape [4–6]. Various hormones such as insulin, cortisol, leptin, and ghrelin, which are critical regulators of our metabolism, might undergo changes during this fasting period. Similarly, the fasting state can also impact key biochemical parameters, including glucose, lipid, and protein metabolism [7, 8].

Several individual studies have looked into these effects, with some suggesting significant changes, while others report minimal or no impacts. The rationale for our systematic review rests on its broader analytical lens, examining both major endocrine hormones and key biochemical parameters. While we acknowledge and have cited existing reviews that focus on specific aspects such as glucometric markers [9], our work aims to offer a more comprehensive assessment of the metabolic and hormonal changes associated with Ramadan fasting in healthy non-athlete adults. In line with the PICOS framework, our research objective is: (P) Healthy non-athlete adults; (I) Observing Ramadan fasting; (C) Measurements before the onset of Ramadan (pre-fasting values); (O) Changes in serum levels of T3, T4, TSH, FT3, FT4, Testosterone, LH, FSH, Prolactin, PTH, Calcium, Phosphorus, and morning cortisol; (S) Observational studies, case-control studies, and quasi-controlled studies. Through this review, we aim to discern whether and to what extent Ramadan fasting influences these critical endocrine parameters in the specified population.

## Material and methods

### Ethical approval

The study protocol was reviewed and accepted by the ethics committee of the Endocrinology and Metabolism Research Institute of Tehran University of Medical Sciences (ID: IR.TUMS.EMRI.REC.1395.00143).

### Registration

The protocol of this systematic review was registered a priori in PROSPERO (CRD42020135123).

### Database searches

This systematic review and meta-analysis was conducted according to the Preferred Reporting Items for Systematic reviews and Meta-Analyses (PRISMA) guidelines [10].

### Search strategy

A comprehensive literature search was carried out on the Web of Science, PubMed, Scopus, Cochrane Library, and Embase databases in September 2023. The search focused on full published papers in any language. Given the nature of Ramadan fasting, studies evaluated hormonal and biochemical changes before and after Ramadan; therefore, only observational studies were included. Our search strategy combined keywords such as: "Fasting", “Ramadan”, and "Hormones" along with their Medical Subject Headings (MeSH) terms. All retrieved papers were imported into Endnote Reference Manager. Duplicates were identified and excluded using the “remove duplicates” function and manual checks. Additional articles were sourced by hand-searching reference lists of included articles and using Google Scholar.

### Study selection

Three reviewers (SHM and MP and JSH) independently screened the titles and abstracts of identified studies and full-text reports of eligible studies were obtained for data extraction. Where there was a lack of consensus an expert consultation was performed.

### Inclusion criteria

Population (P): Healthy adults without limitations regarding gender, ethnicity, and geographical area.

Intervention (I): Observing the effects of Ramadan fasting on the parameters of interest.

Comparison (C): Changes in the parameters of interest before (baseline—a few days before or the first day of Ramadan) and after the completion of Ramadan or at least 2 weeks into the fasting month.

Outcome (O): Levels of hormones/biochemical parameters of interest.

Study design (S): Observational studies published in peer-reviewed journal of any language.

### Exclusion criteria

Studies on special populations like athletes, pregnant or lactating women, and patients with different illnesses.

Studies using specimens other than serum for parameter evaluations (e.g., serum vs. salivary for cortisol).

Studies providing results solely through graphs without numerical data.

Conference abstracts, case reports, editorials, review articles, letters, animal studies, and unpublished papers (since they did not undergo peer-review process).

Studies that are published in predatory journals according to Bell’s list

Inaccessibility of the full text even after contacting the corresponding author thrice via email.

### Grouping for syntheses

By Parameter of Interest: Studies will further be grouped based on the specific hormone or biochemical parameter they are analyzing.

### Main outcomes and measures

Main outcome measures consisted of the mean changes in serum levels of parameters of interest in terms of before Ramadan, during Ramadan (if applicable), end of Ramadan, and post Ramadan (if applicable): TSH, T4, T3, LH, FSH, testosterone, cortisol, prolactin, PTH, calcium, and phosphorous. Among the included studies these parameters were the main frequently used outcome that were available.

### Data extraction

Data extraction was performed by two mentioned investigators. Information extracted from articles was first author name, year of published study, design, participants’ characteristics (sample size, gender, and age, fasting time and place, duration of fasting (hours) and time measures of hormones.

### Quality assessment of studies

The JBI (Joanna Briggs Institute) Critical Appraisal Checklist was used by three reviewers independently to appraise the methodological quality of the included studies [11, 12]. Bias was appraised through nine questions that aim to evaluate the research design of the study followed by the similarities of participants in any comparison and the type of care they received checklists consist of items to assess selection, the existence of a control group, the existence of multiple measurements, follow‐up description, the existence of same outcome measurement, reliability of outcome measurements, and the appropriate statistical analysis used. Each criterion had four possible responses including “Yes,” “No,” “Unclear,” or “not applicable. JBI checklist is not a scoring system for grading the studies as well as lack of any consensus about cut-off point for defining the inclusion of the study [13]. (Table 1)

**Table 1:** Summary of the quality appraisal for the included studies using the Joanna Briggs Institute (JBI) Critical Appraisal Checklist.

	Author, year	the ’cause’ and is the ‘effect’ clearly defined	Similarity of the participants included in any comparisons	receiving similar treatment/care in any participants	multiple measurements of the outcome	Completeness of follow-up	Outcome measured in the same way	Reliability of outcomes measured	appropriate statistical analysis using
1	Fedail, S. S. 1982 [16]	+	N/A	N/A	+	+	N/A	+	+
2	Abbas, S. M. 1986 [17]	+	N/A	N/A	+	+	N/A	+	+
3	Azizi, F 1987 [18]	+	N/A	N/A	+	+	N/A	?	+
4	Sulimani, R. A. 1988 [19]	+	N/A	N/A	+	+	N/A	+	+
5	Azizi, F 1991 [20]	+	N/A	N/A	+	+	N/A	+	+
6	Sajid, KM,1991 [21]	+	N/A	N/A	+	+	N/A	+	+
7	Bakir, SM, 1992 [22]	+	N/A	N/A	+	+	N/A	+	+
8	Azizi F, 1994 [24]	+	N/A	N/A	+	+	N/A	+	+
9	Bakir, 1994 [25]	+	N/A	N/A	+	+	N/A	+	+
10	Elati, J. 1995 [27]	+	N/A	N/A	+	+	N/A	+	+
11	Aybak, 1995 [26]	+	N/A	N/A	+	+	N/A	+	+
12	Dwivedi, S. 1996 [28]	+	N/A	N/A	+	+	N/A	?	+
13	Iraki, 1997 [7]	+	N/A	N/A	+	+	N/A	+	+
14	Bilto, Y. Y. 1998, [29]	+	N/A	N/A	+	+	N/A	+	+
15	Mira, S. A. 2002, [30]	+	N/A	N/A	+	+	N/A	+	?
16	Ben salem, 2003 [23]	+	N/A	N/A	+	+	N/A	+	+
17	Shahrzad, M 2003 [31]	+	N/A	N/A	+	+	N/A	+	+
18	El-Migdadi, F. 2004 [32]	+	N/A	N/A	+	+	N/A	+	+
19	Mesbahzadeh, B 2005 [1]	+	N/A	N/A	+	+	N/A	+	+
20	Ahmadinejad, Z. 2006 [5]	+	N/A	N/A	+	+	N/A	?	+
21	Ghaderi,2006 [33]	+	N/A	N/A	+	+	N/A	+	+
22	Ghiravani, Z 2006 [34]	+	N/A	N/A	+	+	N/A	+	+
23	Marbut, 2006 [35]	+	N/A	N/A	+	+	N/A	+	+
24	Mansi, K. 2007 [36]	+	N/A	N/A	+	+	N/A	+	+
25	Shahabi, 2010 [37]	+	N/A	N/A	+	+	N/A	+	+
26	Sülü, B. 2010 [38]	+	N/A	N/A	+	+	N/A	+	+
27	Çağlayan, 2012 [39]	+	N/A	N/A	+	+	N/A	+	+
28	Bahijri, S., A, 2013 [40]	+	N/A	N/A	+	+	N/A	+	+
29	Sayedda, K. 2013 [41]	+	N/A	N/A	+	+	N/A	+	+
30	Al Nahari,2014 [42]	+	N/A	N/A	+	+	N/A	+	+
31	Bahijri, S., A, 2015 [43]	+	N/A	N/A	+	+	N/A	+	+
32	Talib, R. A. 2015 [44]	+	N/A	N/A	+	+	N/A	?	+
33	Sedaghat, MR. 2017 [45]	+	N/A	N/A	+	+	N/A	+	+
34	Papazoglou, 2020 [46]	+	N/A	N/A	+	+	N/A	+	+
35	Riat, 2021 [47]	+	N/A	N/A	+	+	N/A	+	+

(“+” = “yes,” “−” = “no,” "N/A" = not applicable and “?” = “unclear”)

### Strategy for data synthesis

#### Qualitative literature review

The evidence of changes in hormonal levels and biochemical parameters during Ramadan Fasting were narratively synthesized, separately. However, the conclusion from the narrative review was planned to be validated by conducting a subsequent meta-analysis.

#### Quantitative literature review (meta-analysis)

Continuous variables were presented as means and standard deviations (SD). Whenever an article reported standard error (SE) instead of standard deviation, standard deviations were calculated based on the formula association among them (SE = SD/√ n). Categorical (binary) data were reported as n (%). whenever it was possible to conduct a meta-analysis, it was performed with STATA software, version 17(Stata Corp, USA).

Heterogeneity Assessment: The Chi-square-based *Q*-test was used to assessing heterogeneity and was considered to be statistically significant at *P* < 0.1. The degree of heterogeneity was estimated using I^2^ statistic. In overall, the I^2^ value of 25% was considered low, the I^2^ value of 50% was considered as moderate, and the I^2^ value of 75% and more was considered as high heterogeneity [14]. Due to the limited number of included studies in each group, a deeper exploration of the sources of heterogeneity was not feasible. Specifically, methodologies such as subgroup analysis, meta-regression, and sensitivity analyses were not conducted due to the insufficient quantity of studies, making these analyses less meaningful and potentially misleading.

Effect Size Measurement: The Fixed-effect model was performed, when no significant statistical heterogeneity between studies were detected (P>0.2), a in case of presenting any heterogeneity random-effects model was used (p≤0.2). Hedges’ g value was used for effect size measurement and also forest plots were used to present the results graphically and to illustrate point estimates of the effect size and 95% confidence interval (CI). An effect size of ≤0.2 was described as a small effect, an effect size around 0.5 as a medium effect, and an effect size around 0.8 was as a large effect [15]. If Hedges’ g CI did not contain 0, In addition to Hedges’ g values and the mean difference’s forest plot is illustrated.

Publication Bias: Funnel plots and Egger’s test were applied to detect potential publication biases.

## Results

### Characteristics of studies

**Fig 1:**
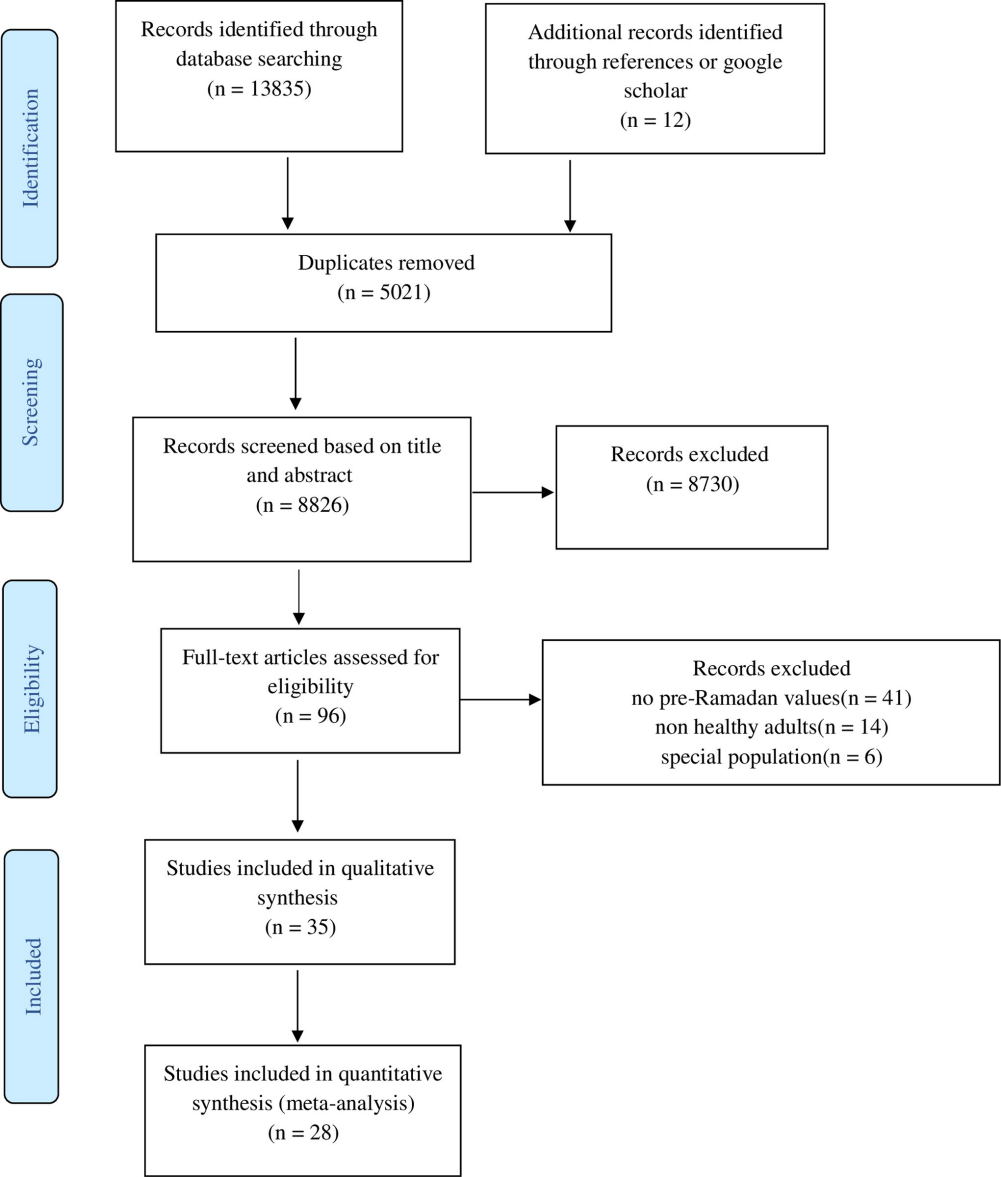
PRISMA flow diagram for the selected studies.

Through searches across multiple databases and other resources, we identified 13,085 papers relevant to our search strategy. After eliminating duplicate documents, we assessed the titles and abstracts of 8,826 publications, which culminated in the full-text retrieval of 96 papers. Eventually, we included 35 publications (encompassing 1,107 participants: 783 men, 271 women, and 53 unknown gender) in our final analysis (Fig 1) [1, 5, 7, 16–47]. The included studies spanned between 1982 and 2021. Detailed information quality assessment of studies can be found in Table 1. Notably, no studies were removed due to quality assessment.

Among the selected studies, nineteen focused solely on men, four exclusively on women, while twelve reported results for both genders (Table 2). Two studies did not specify the participant number by gender. The mean age of included participants was 27.99 ± 6.17 years, ranging from 17–56. All the studies implemented a pre-post design to report changes in hormones and/or biochemical markers. Notably, none of the studies included a control group of non-fasted participants.

**Table 2:** Baseline characteristic of the included studies in the systematic review.

	First Author (year of publication)	location of study	Sample size /Gender	Season of fasting	mean age(age range),years	mean fasting duration(hours)	fasten days	Hormones/biochemical parameters examined	measuring times (relative to the Ramadan fast)	Results (after compared with before Ramadan months)
1	Fedail, S. S. 1982 [16]	Khartoum, Sudan	Overall: 24 F:4 M:20	NR	30(21–40)	16	NR	total T4, total T3,	1^st^ and last day	• Significant rise in serum total T4 • No significant changes in total T3
2	Abbas, S. M. 1986 [17]	Jeddah, Saudi Arabia	M:8	NR	NR	NR	NR	LH, FSH, PRL, Testosterone	Before fast, end of fast, post fast	• Significant increase in LH, and FSH • nonsignificant elevation in testosterone
3	Azizi, F 1987 [18]	Tehran, Iran	M:9	Summer	35(23–54)	17	29	calcium	7 days before, 29 ^th^ day, 28 days after	nonsignificant drop in calcium
4	Sulimani, R. A. 1988 [19]	Saudi Arabia	M:28	-	25–50	14.5	30	TSH, T3, total T4, free T4	1^st^ day/30 ^rd.^ day	no significant changes in the thyroid function tests
5	Azizi, F 1991 [20]	Tehran, Iran	M:9	Summer	35(23–54)	17	29	FSH, LH, testosterone, T4, T3, TSH, PRL	7 days before, 29^th^ day, 28 days after	no significant changes in the thyroid function tests, sex hormones, and PRL
6	Sajid, KM,1991 [21]	Pakistan	Overall: 46 M:41 F:5	Spring	28–56	16	29	TSH, total T4, total T3	20 days before,26th day,23 days after	• significant rise in TSH • no significant change in total T4 or total T3
7	Bakir, SM, 1992 [22]	Saudi Arabia	M:11	NR	24–46 34.9±1.6	14		FSH,LH	Before fasting, during fasting	Significant increase in 4a.m during fasting in FSH and LH
8	Azizi F, 1994 [24]	Tehran, Iran	F:12	-	20–25	16	-	Total T4, total T3	1^ST^ day, 29^th^ day, 10 days after	• Significant decrease in T4 and T3
9	Bakir, 1994 [25]	Saudi Arabia	M:9	NR	40.11(5.37) 27–48	14		calcium, PTH	Before fasting, during fasting	Significant decrease in calcium
10	Elati, J. 1995 [27]	Tunisia	F:16	-	25–39	13	-	calcium, phosphorous, cortisol	2 days before, 28^th^ day, 28 days after	No significant change in calcium and phosphorous • nocturnal rise in cortisol
11	Aybak, 1995 [26]	Turkey	M:22	NR	28 (4.2)	16	30	Cortisol, GH	Baseline, 1^st^ day, 14^th^ day, 28^th^ day	Significant increase in cortisol
12	Dwivedi, S. 1996 [28]	India	M:12	-	18–25	-	28	cortisol	1 day before, 28^th^ day	Significant decrease in morning cortisol level
13	Iraki, 1997 [7]	Morocco	M:9		25 (1.2), 20-32y	NR	NR	calcium	once before Ramadan, 24^th^ day, 1 month after the end of Ramadan	Significant decrease in calcium
14	Bilto, Y. Y. 1998, [29]	Jordan	33	1997	20–48	11*	28	calcium, phophorous	1–7 days before, day 21–28	No significant change in calcium and phosphorous
15	Mira, S. A. 2002, [30]	Saudi Arabia	Overall: 58 F:10 M:48	-	37.05±9.87 F: 28.9±5.15 M:38.75 ±9.79 (19–56)	-	-	testosterone	1 day before, 21^st^, 14 days after	• M: no significant change in testosterone F: Significant decrease in testosterone
16	Ben salem, 2003 [23]	Tunisia	M: 11	December 2000	20–35 (26.5 ± 1.4)	11.5	16–22 (average 17 d)	Cortisol (a.m., p.m.)	Before Ramadan, during Ramadan	No significant change
17	Shahrzad, M 2003 [31]	Iran	Overall:98 F:46 M:52	Autumn	20.6±3.3	11.5	30	TSH,TT4,TT3, freeT4,freeT3	1^st^ day, 28^th^ day	Overall: • Significant decrease in total T4 and total T3 • no significant change in TSH, free T4 or free T3 M: • Significant decrease in total T4, total T3, TSH, and freeT4 • no significant change in free T3 F: • Significant decrease intotal T3 • no significant change in total T4, TSH, free T3, and freeT4
18	El-Migdadi, F. 2004 [32]	Jordan valley and Ramtha city, Jordan	M:40	Winter	17–25	12		LH, testosterone	before fast, end of fast	no significant change in LH or testosterone
19	Mesbahzadeh, B 2005 [1]	Birjand, Iran	M:52	winter	18–24	12	28	Testosterone, LH, FSH	2 days before, 28^th^ day	• significant decrease in total testosterone • no significant change in FSH or LH
20	Ahmadinejad, Z. 2006 [5]	Tehran, Iran	80 F:39 M:41	Winter	22.7 ±2.3(18–29)	12	more than 15 days	TSH, T3, T4	3 days before, day 26	Overall: • Significant decrease in total T4• no significant change in total T3 • significant increase in TSH M: • Significant decrease in total T4 • no significant change in total T3 • significant increase in TSH F: • Significant decrease in total T • no significant change in total T3 or TSH
21	Ghaderi,2006 [33]	Iran	M:32		26 ± 2 (24–28)	12	28	LH, FSH, Testosterone	1 day before, 28^th^ day	• significant increase in testosterone • significant decrease in FSH • no significant change in LH
22	Ghiravani, Z 2006 [34]	Birjand, Iran	M:52	NR	18–24	12	28	T4, T3, TSH	2 days before, 28^th^ day	• significant increase in total T4 • no significant change in total T3 or TSH
23	Marbut, 2006 [35]	Tikrit, Iraq	20	Autumn	NR	12	NR	PTH, calcium, phosphorus	pre-Ramadan, end of Ramadan	• significant increase in PTH and calcium • no significant change in phosphorus
24	Mansi, K.2007 [36]	Jordan	M:42	Autumn	21.3±1.6	12	NR	testosterone, LH, FSH, total T3, total T4	1 day before, day 21–28, 14 days after	• Significant decrease in testosterone • significant increase in FSH • nonsignificant decrease in total T4 • no significant changes in total T3 or LH
25	Shahabi, 2010 [37]	Iran	F: 24	2008	20.45 (0.99)	11	23–24	FSH, LH, Estradiol	Ramadan, 2 months later	• no significant changes FSH or LH
26	Sülü, B. 2010 [38]	Turkey	Overall:45 F:22 M:23	Autumn	21-51(28.7) M:30.5±7.1 F:26.9±3.8	13.5*	NR	TSH, FT3, FT4	1 day before/28^th^ day	Overall: • Significant decrease TSH • no significant change in free T4 or free T3 M: • Significant decrease free T4 • no significant change in TSH or free T3 F: • no significant change in TSH, free T4 or free T3
27	Çağlayan, 2012 [39]	Turkey	F: 30	Summer,2011	21–41	14.5	21–24	FSH, LH, testosterone, PRL	1–2 months before, during Ramadan	No significant change in FSH,LH, testosterone, PRL
28	Bahijri, S., A, 2013 [40]	Saudi Arabia	Overall:23 F:5 M:18	N/R	18-42/ 23.1 ± 1.2	NR	NR	cortisol	before and 10–15 days in to Ramadan	No significant change in morning cortisol • significant rise in PM cortisol
29	Sayedda, K. 2013 [41]	India	M: 20	Summer	19–32	15	29	calcium, phosphorous	2 days before, 29^th^ day	No significant change in calcium and phosphorous
30	Al Nahari,2014 [42]	Saudi Arabia	M:26	NR	NR	NR	NR	Cortisol, Testosterone, TSH, FT4 and FT3	During fast/post fast	• nonsignificant increase in cortisol • no significant change in testosterone • significant decrease in TSH • significant increase in free T4, and free T3
31	Bahijri, S., A, 2015 [43]	Saudi Arabia	Overall: 23 F:5 M:18	NR	18-42/ 23.1 ± 1.2	NR	NR	PTH calcium, phosphorus	before and 2 weeks in to Ramadan	No significant change in calcium and phosphorous
32	Talib, R. A. 2015 [44]	Doha, Qatar	M:45	NR	27-56/37 ± 7.2	NR	30	total T, free T, LH, FSH, Estradiol	1 week before/ 29^th^ or 30^th^ of month	• no significant change in testosterone or LH • significant decrease in FSH
33	Sedaghat, MR. 2017 [45]	Mashhad, Iran	Total: 89 F:38 M:51	Summer	34.52± 9.05 (20–50)	17	At least 20 days	Calcium, phosphorous	1 week before/1 week after	• No significant change in calcium • significant reduction in phosphorous (total and males)
34	Papazoglou, 2020 [46]	Greece	M: 15	Spring 2020	21.1 ± 0.9	NR	30	Calcium, morning cortisol	1 day before/30^th^ day	• No significant change in calcium • Significant decrease in morning cortisol
35	Riat, 2021 [47]	Germany	Total: 34 F:15 M: 19		25.1 ± 0.8	NR	29	Cortisol	1 week before mid of RF last days of RF, 1 week after RF, and 1 month after RF (T5)	Significant decrease in morning cortisol

Interestingly, one study investigated the effects of Ramadan fasting on two different regions in Jordan [32]. The mean fasting hours reported across 28 studies ranged from 11 to 17 hours (13.44±1.97 hours).

We categorized the effects of Ramadan fasting according to the hormonal and biochemical markers studied: Thyroid hormones, Cortisol, Sex hormones, and Calcium, Phosphorous, and Parathyroid hormone (PTH).

### Effect of Ramadan on thyroid hormones

**Fig 2:**
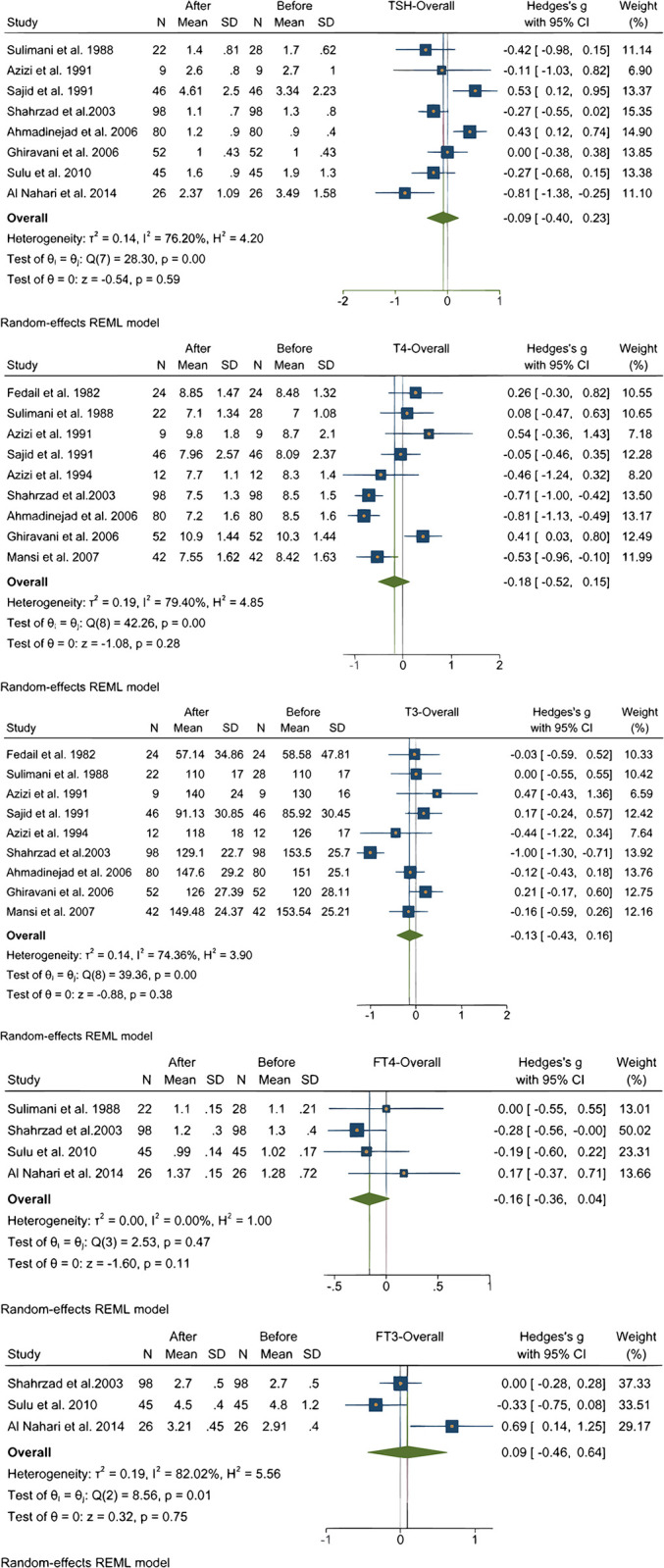
Effect of Ramadan fasting on thyroid hormones.

In overall 11 studies assessed the changes of Thyroid hormones (TSH, T3, AND T4) during Ramadan fasting [5, 16, 19–21, 24, 31, 34, 36, 38, 42]. Analysis of pooled data of participants did not show a significant change in TSH [5, 19–21, 24, 31, 34, 38, 42], Total T4 [5, 16, 19–21, 24, 31, 34, 36], Total T3 [5, 16, 19–21, 24, 31, 34, 36], Free T4 [19, 31, 38, 42], and Free T3 [31, 38, 42] levels as it is shown in Fig 2. There was no significant difference between female and male participants regarding TSH, Total T4, Total T3, Free T4, and Free T3 levels. Heterogeneity of studies were high regarding TSH, Total T4, Total T3, and Free T3 levels. Heterogeneity of studies with Free T4 levels were low (*I*^2^ = 0.0%, *H*^2^ = 1.0); We repeated meta-analysis using inverse-variance fixed-model which its result did not differ from random-model.

### Effect of Ramadan on cortisol

Three studies evaluated changes in cortisol levels during 24-hours before and after Ramadan fasting. They all found a diurnal decrease in cortisol levels and nocturnal increase in serum cortisol levels during Ramadan fasting [27, 48, 49].

Two studies evaluated cortisol in the morning and in the evening [28, 40]. Dwivedi et al. [28] reported a significant reduction in morning cortisol levels, and significant rise in evening cortisol levels compared to pre-Ramadan values in 12 young healthy Muslims men. However, Bahijri et al. [40] did not observe any significant change in morning cortisol values (p = 0.06), but significant rise in evening cortisol values (p = 0.01) two weeks in to Ramadan in 23 young Ramadan practitioners compared to pre-Ramadan cortisol values.

**Fig 3:**
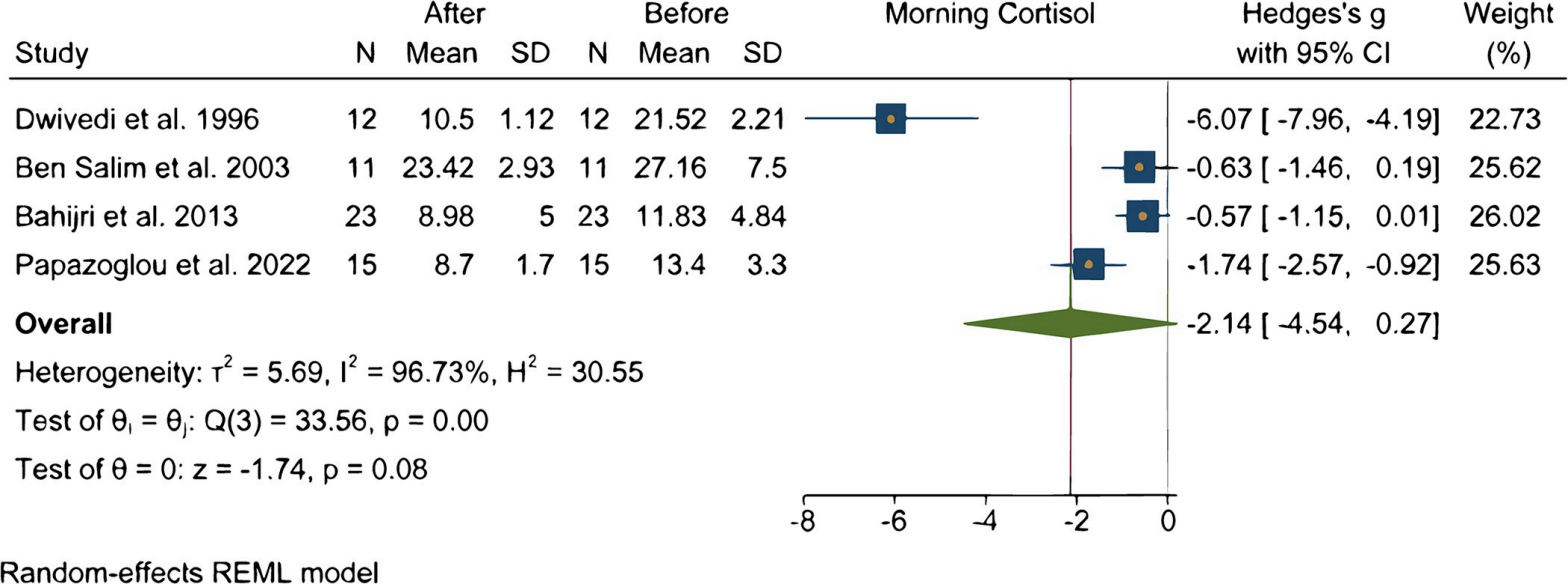
Changes in cortisol levels during Ramadan fasting.

Four studies evaluated the impact of Ramadan fasting on morning cortisol levels [23, 28, 40, 46]. Analysis of pooled data of participants showed a large insignificant decrease (P = 0.08) in morning cortisol levels as it is shown in Fig 3.

### Effect of Ramadan fasting on sex hormones

**Fig 4:**
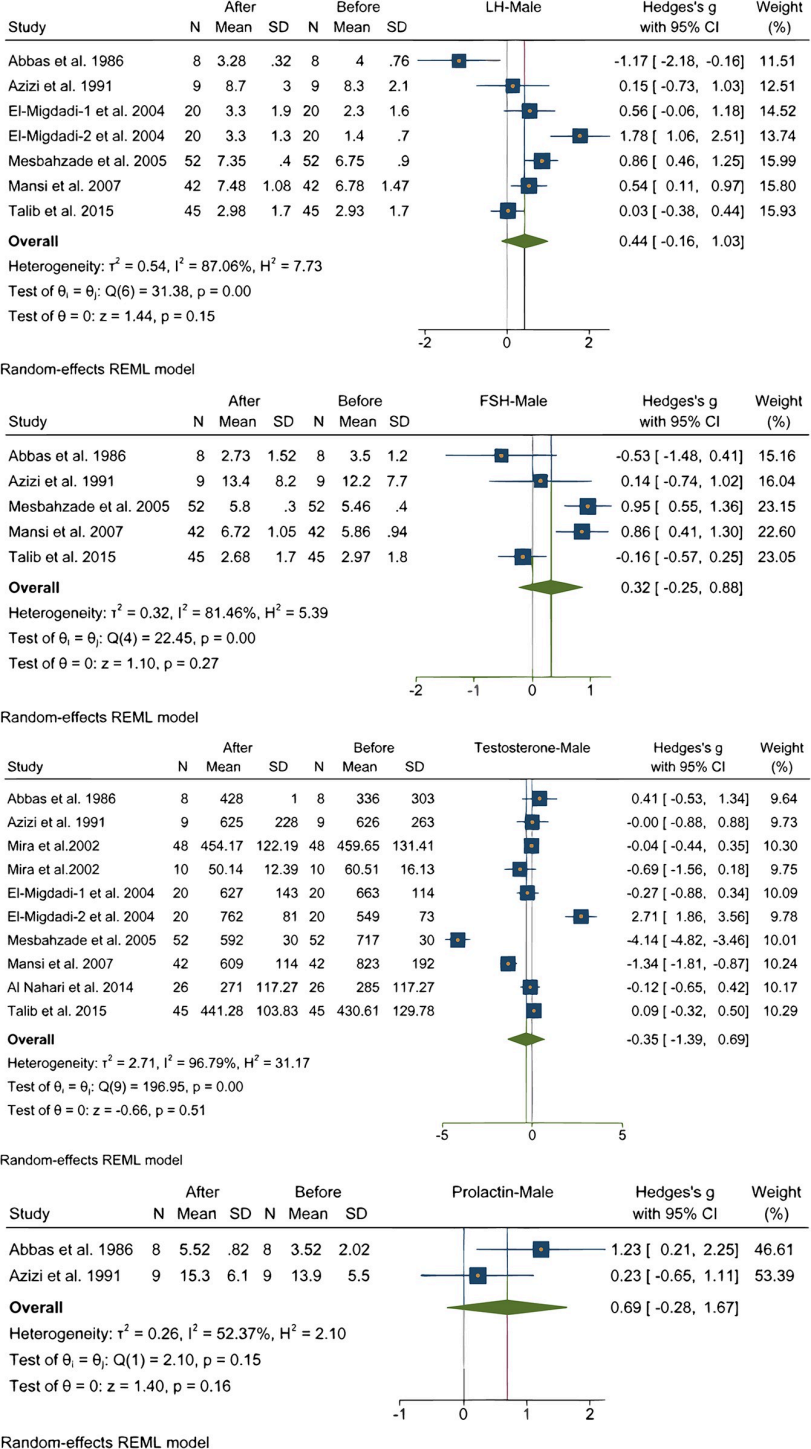
Impact of Ramadan fasting on sex hormones.

In overall 8 studies assessed the changes of Sex hormones (Testosterone, LH, FSH) among men during Ramadan fasting. Analysis of pooled data of participants did not show a significant change in Testosterone [1, 17, 20, 30, 32, 36, 42, 44], LH [1, 17, 20, 32, 36, 44], FSH [1, 17, 20, 36, 44], Prolactin [17, 20] levels as it is shown in Fig 4. Heterogeneity of studies were high regarding Testosterone, LH, FSH, Prolactin levels.

El-Migdadi et al. [32] assessed serum LH and testosterone values pre- and post- Ramadan fasting in two groups healthy male individuals from two different regions of Jordan country. They found that fasting caused a significant increase in serum LH and testosterone values only in subjects of in the below sea level environment.

One study by Shahabi et al. evaluated the effect of Ramadan fasting on LH, FSH levels and Ovulation in females; and concluded that Islamic fasting causes neither significant variation in the secretion of hormones around ovulation nor does it influence the occurrence of ovulation [37].

### Effect of Ramadan fasting on calcium, phosphorous, and PTH

**Fig 5:**
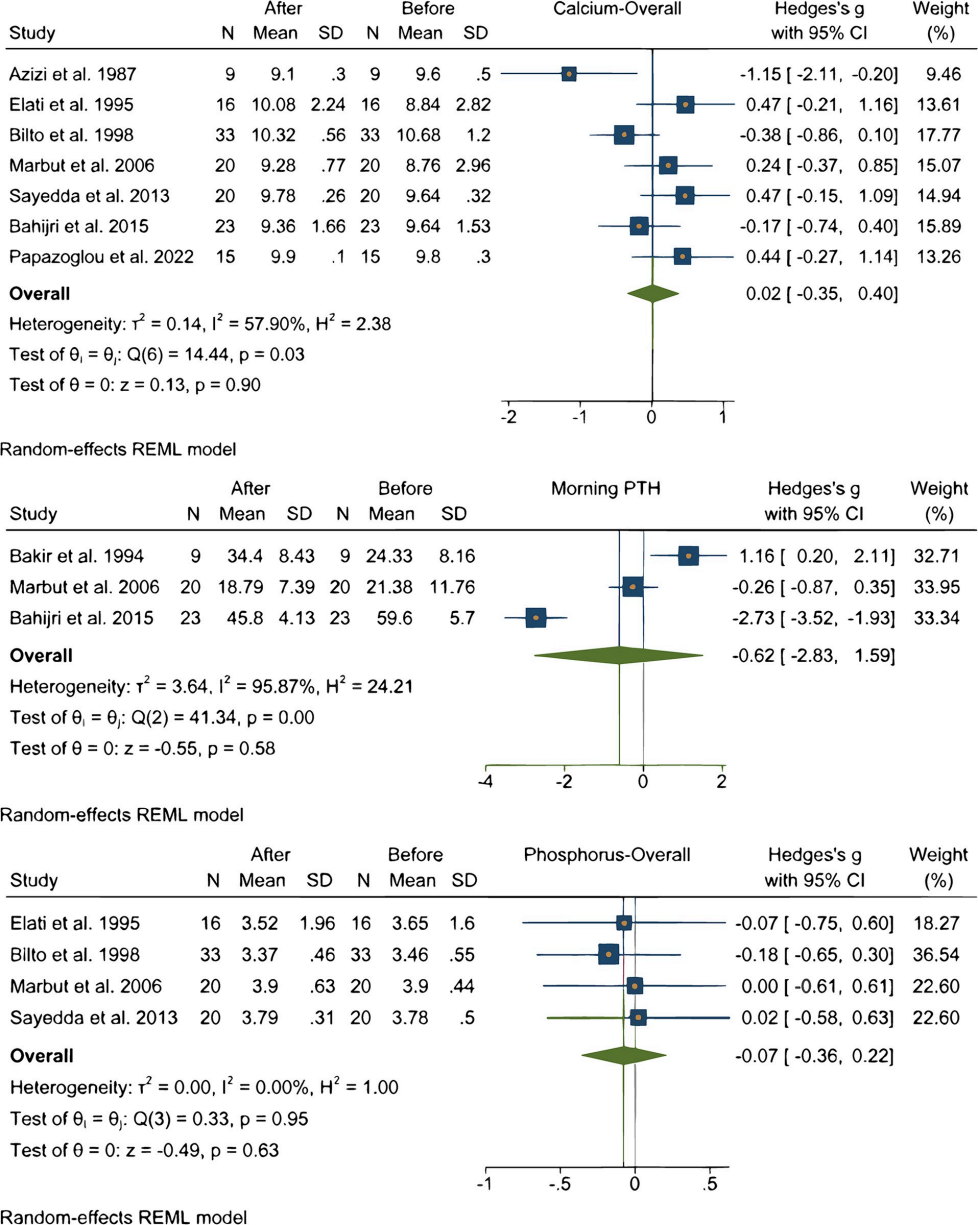
Effects of Ramadan fasting on calcium, phosphorous, and PTH levels.

In overall 8 studies assessed the changes of Calcium, phosphorous, PTH during Ramadan fasting [18, 25, 27, 29, 35, 41, 43, 46]. Analysis of pooled data of participants did not show a significant change in Calcium [18, 27, 29, 35, 41, 43, 46], phosphorous [27, 29, 35, 41], PTH [25, 35, 43] levels as it is shown in Fig 5. Heterogeneity of studies were high regarding Calcium, phosphorous, PTH levels.

## Discussion

The objective of our systematic review and meta-analysis was to elucidate the impact of Ramadan fasting on the serum levels of major endocrinology hormones and biochemical parameters in healthy non-athlete adults. Our analysis incorporated data from a diverse array of studies, capturing variations in geographical location, gender, age, and fasting duration.

We found no significant changes in thyroid hormones (TSH, T3, T4), sex hormones (Testosterone, LH, FSH, Prolactin), and key biochemical markers (Calcium, Phosphorous, PTH) during Ramadan fasting across genders. However, we did observe a trend towards diurnal decrease and nocturnal increase in serum cortisol levels. While these findings were not statistically significant, they may still suggest physiological adaptations to the altered eating and sleeping patterns during Ramadan.

Our findings are in alignment with the notion that intermittent fasting during Ramadan does not induce drastic changes in endocrine functions or biochemical parameters in healthy individuals. This could potentially be attributed to the adaptive capacity of the human body, which has evolved mechanisms to maintain metabolic and hormonal homeostasis even during periods of fasting.

Ramadan fasting represents a well-recognized model of religious intermittent fasting that significantly alters eating and sleep patterns for an entire month. These changes could potentially disrupt the normal functioning of various body organs. However, several researchers have suggested that intermittent fasting may indeed have beneficial effects on human health [50, 51]. Numerous original research studies, systematic reviews, and meta-analyses have been conducted to evaluate the effects of Ramadan fasting on various health-related factors in both healthy and non-healthy individuals. It is important to note that as the Islamic world spans across diverse geographical locations, featuring different dietary patterns and social habits, the fasting effects observed in Ramadan practitioners from various regions are likely to differ [27].

During Ramadan, alterations in the sleep-wakefulness cycle and changes in social habits might potentially affect the circadian pattern of certain hormonal parameters. These include pituitary hormones (PRL, LH, FSH, GH, and TSH), steroid hormones (cortisol, testosterone), and thyroid hormones [52]. Our study found that Ramadan Intermittent Fasting could result in a considerable yet statistically insignificant decrease in Morning Cortisol levels. Furthermore, most of the reviewed studies reported a significant rise in Evening Cortisol levels. The consensus from these studies was that these changes were largely due to alterations in circadian rhythm during Ramadan.

The findings regarding changes in thyroid hormones during Ramadan were somewhat mixed. According to the results of included studies, Ramadan fasting does not appear to adversely affect thyroid function. Any observed changes in thyroid-related hormones remained within normal limits during this month.

### Effect of Ramadan fasting on glucometabolic markers

Our study initially intended to conduct a meta-analysis on glucometabolic markers. However, during our review process, an exhaustive systematic review by Faris et al. was published [9]. In light of their recent work, we instead performed a systematic update for newer studies on glucometabolic markers. Three new studies observed a minor rise in blood sugar levels (one of which was statistically insignificant) [53–55]. Additionally, one study reported a slight and insignificant rise in serum insulin levels [53]. None of these findings contradicted the previously mentioned systematic review.

Our meta-analysis revealed no significant changes in TSH, total T4, and total T3 during Ramadan. A significant reduction was, however, observed regarding free T4 values. In women, total T4 and T3 hormone concentrations may decrease due to alterations in binding proteins in the last days of Ramadan, but free hormone indices remain within normal limits [5, 24].

### Limitations

It is crucial to highlight the high heterogeneity observed for several parameters, which could be due to methodological differences across studies, such as the timing of sample collection, the participant’s age, gender, and lifestyle factors. This heterogeneity underscores the need for standardized protocols in future studies to ensure comparability and consistency of findings. Despite the lack of a control group of non-fasted participants in the included studies, our review still offers valuable insights into the physiological adaptations during Ramadan fasting. It should be noted, though, that our findings may not apply to specific populations such as athletes, elderly, or those with underlying health conditions, and therefore caution must be exercised when generalizing the findings.

## Conclusion

This meta-analysis findings suggest that Ramadan fasting has not any adverse effect on glucometabolic markers, sexual hormones, thyroid hormones, calcium, phosphorus, and PTH. There was a near significant decrease of serum morning cortisol level, which it is due to the change in circadian rhythm. Ramadan Intermittent Fasting imposes minimum hormonal change, and is a safe practice.

## Supporting information

S1 Checklist PRISMA 2020 checklist. (DOCX)

S1 Data (ZIP)
