# Supplementary material for: Impact of Ramadan fasting on serum levels of major endocrinology hormonal and biochemical parameters in healthy non-athlete adults: A systematic review and meta-analyses
Source: PLoS One. 2024 May 23;19(5):e0299695. doi: 10.1371/journal.pone.0299695 (PMC11115274; doi:10.1371/journal.pone.0299695)
Supplement: S1 Data — (ZIP) [file pone.0299695.s002.zip › Supplements/search strategy (1).docx]

SEARCH DATE: 15 September 2022

**PubMed:**

("Fasting"[Mesh] OR fasting[tiab] OR fatings[tiab]) AND (Ramadan OR Ramedan OR Ramezan OR ramazan OR Ramdan OR Ramzan OR religious OR Islamic OR intermittent ) AND ("Hormones"[Mesh] OR Hormone OR Hormones OR Prolactin OR "Prolactin"[Mesh] OR Mammotropin OR PRL OR "Somatostatin"[Mesh] OR “SRIH-14” OR “Somatostatin-14” OR [Somatostatin](http://www.ncbi.nlm.nih.gov/mesh/68013004) OR Pituitary OR HypophyS* OR (Infundibular AND (Stalk* OR  Stem)) OR Infundibulum* OR Corticoliberin OR CRF-41 OR "Corticotropin-Releasing Hormone"[Mesh] OR CRF OR (((Corticotropin OR ACTH ) AND Releasing) AND (Factor OR Hormone)) OR CRH OR "Gonadotropin-Releasing Hormone"[Mesh] OR ([Gonadotropin-Releasing AND Hormone](http://www.ncbi.nlm.nih.gov/mesh/68007987)) OR GnRH OR LFRH OR Gn-RH OR LHFSHRH OR LHRH OR LH-RH OR "Growth Hormone-Releasing Hormone"[Mesh] OR ([Growth AND Hormone-Releasing AND Hormone](http://www.ncbi.nlm.nih.gov/mesh/68013007)) OR GHRH OR “GRF 1-44” OR Somatocrinin OR Somatoliberin OR “GHRH 1-44” OR “hpGRF 44” OR ([Thyrotropin-Releasing AND Hormone](http://www.ncbi.nlm.nih.gov/mesh/68013973)) OR TRH OR Stimu-TSH OR “Stimu TSH” OR “StimuTSH” OR [Pro-Opiomelanocortin](http://www.ncbi.nlm.nih.gov/mesh/68011333) OR "Pro-Opiomelanocortin"[Mesh] OR “Pro Opiomelanocortin” OR Proopiomelanocortin OR Pro-“ACTH-Endorphin” OR “Pro ACTH Endorphin” OR “Pro-Opio-Melanocortin” OR “Pro Opio Melanocortin” OR “Pro-Opiocortin” OR “Pro Opiocortin” OR “Proopiocortin” OR “ ACTH-Endorphin Precursor” OR “ACTH Endorphin Precursor” OR “Endorphin-ACTH Precursor” OR “Endorphin ACTH Precursor” OR Preproopiomelanocortin OR “Pre-pro-opiocortin” OR “Pre pro opiocortin” OR “Pre-POMC” OR “Pre POMC” OR Opiocortin OR POMC OR [Gonadotropin*](http://www.ncbi.nlm.nih.gov/mesh/68006065) OR "Follicle Stimulating Hormone"[Mesh] OR “ Follicle-Stimulating Hormone” OR “ [Follicle Stimulating Hormone](http://www.ncbi.nlm.nih.gov/mesh/68005640)” OR FSH OR [Luteinizing](http://www.ncbi.nlm.nih.gov/mesh/68007986)  OR "Luteinizing Hormone"[Mesh] OR LH OR “ICSH Interstitial Cell Stimulating Hormone” OR “[Growth Hormone](http://www.ncbi.nlm.nih.gov/mesh/68013006)” OR “[Growth Hormone](http://www.ncbi.nlm.nih.gov/mesh/68013006)S” OR "Growth Hormone"[Mesh] OR Somatotropin* OR GH OR (Adrenocorticotropic AND Hormone) OR "Adrenocorticotropic Hormone"[Mesh] OR Corticotrophin OR Corticotropin OR “1-39 ACTH” OR Adrenocorticotropin OR ACTH OR “1-24-Corticotropin” OR “1-24-ACTH” OR TSH OR "Adrenal Glands"[Mesh] OR Adrenal OR [Gonad](http://www.ncbi.nlm.nih.gov/mesh/68006066) OR [Gonad](http://www.ncbi.nlm.nih.gov/mesh/68006066)s OR [Thyroid* OR "Thyroid Gland"[Mesh] OR "Gonadotropins"[Mesh] OR](http://www.ncbi.nlm.nih.gov/mesh/68013961) [Endocrin* OR "Endocrinology"[Mesh] OR](http://www.ncbi.nlm.nih.gov/mesh/68004703)  "Ovary"[Mesh] OR Ovaries OR [Ovary](http://www.ncbi.nlm.nih.gov/mesh/68010053) OR [Ovarian](http://www.ncbi.nlm.nih.gov/mesh/68006080) OR [Testis](http://www.ncbi.nlm.nih.gov/mesh/68013737) OR "Testis"[Mesh] OR Testicles OR Testicle OR Testes OR [Leydig](http://www.ncbi.nlm.nih.gov/mesh/68007985) OR (Testicular AND Interstitial AND Cells) OR [Diiodothyronines](http://www.ncbi.nlm.nih.gov/mesh/68004104) OR "Diiodothyronines"[Mesh] OR [Triiodothyronine](http://www.ncbi.nlm.nih.gov/mesh/68014284) OR "Triiodothyronine"[Mesh] OR Liothyronine OR T3 OR T4 OR Tetraiodothyronine OR [Thyroxine](http://www.ncbi.nlm.nih.gov/mesh/68013974) OR "Thyroxine"[Mesh] OR “T3 resin uptake “ OR t3ru OR “T 3 resin uptake” OR “t3 ru” OR “Free t4” OR “Freet4” OR "Thyroid Function Tests"[Mesh] OR “ Thyroid Function Test” OR “Freet3” OR “Total T4 ” OR “TT4” OR “Total T3” OR “TT3” OR Progesterone OR "Progesterone"[Mesh] OR “[alpha-Dihydroprogesterone](http://www.ncbi.nlm.nih.gov/mesh/68004092)” OR "20-alpha-Dihydroprogesterone"[Mesh] OR "5-alpha-Dihydroprogesterone"[Mesh] OR [Hydroxyprogesterone*](http://www.ncbi.nlm.nih.gov/mesh/68006908) OR "Hydroxyprogesterones"[Mesh] OR [Pregnanediol](http://www.ncbi.nlm.nih.gov/mesh/68011276) OR "Pregnanediol"[Mesh] OR [Estradiol](http://www.ncbi.nlm.nih.gov/mesh/68004958)* OR "Estradiol"[Mesh] OR Oestradiol OR "Estrogens"[Mesh] OR Estrogen*OR Testosterone OR "Testosterone"[Mesh] OR "Adrenal Cortex Hormones"[Mesh] OR Corticosteroids OR Corticoids OR “[17-Ketosteroid](http://www.ncbi.nlm.nih.gov/mesh/68015068)” OR "17-Ketosteroids"[Mesh] OR “17 Ketosteroids” OR “17-Oxosteroids” OR “17 Oxosteroids” OR [Androstenedione](http://www.ncbi.nlm.nih.gov/mesh/68000735) OR "Androstenedione"[Mesh] OR “delta-4-Androstenedione” OR (“4-Androstene-3” AND “17-dione”) OR “5 alpha-Androstan-3 alpha-ol-17-one” OR “3-alpha-Hydroxy-5-alpha-Androstan-17-One” OR “3 alpha Hydroxy 5 alpha Androstan 17 One” OR [Androsterone](http://www.ncbi.nlm.nih.gov/mesh/68000738) OR Epiandrosterone OR [Dehydroepiandrosterone](http://www.ncbi.nlm.nih.gov/mesh/68003687) OR "Dehydroepiandrosterone"[Mesh] OR Dehydroisoandrosterone OR DHEA OR Androstenolone OR “5-Androsten-3-ol-17-one” OR “5 Androsten 3 ol 17 one” OR [Estrone](http://www.ncbi.nlm.nih.gov/mesh/68004970) OR Folliculin OR [corticoids](http://www.ncbi.nlm.nih.gov/mesh/68005938) OR "Glucocorticoids"[Mesh] OR Glucocorticoid OR [Hydroxycorticosteroid*](http://www.ncbi.nlm.nih.gov/mesh/68006889) OR "Hydroxycorticosteroids"[Mesh] OR [Desoxycorticosterone](http://www.ncbi.nlm.nih.gov/mesh/68003900) OR "Desoxycorticosterone"[Mesh] OR Desoxycortone OR Cortexone OR Deoxycorticosterone OR “11-Decorticosterone” OR “21-Hydroxyprogesterone” OR “21 Hydroxyprogesterone” OR [Pregnenolone](http://www.ncbi.nlm.nih.gov/mesh/68011284) OR "Pregnenolone"[Mesh] OR “5-Pregnen-3-beta-ol-20-one”

OR "Blood Glucose"[Mesh] OR (Blood AND (Sugar OR Glucose)) OR "Glucagon"[Mesh] OR Proglucagon OR “HG Factor” OR “Hyperglycemic Glycogenolytic Factor” OR Glucagon OR Glukagon OR [Calcium](https://www.ncbi.nlm.nih.gov/mesh/68002118) OR "Calcium"[Mesh] OR “Factor IV”

OR "Insulin-Like Growth Factor I"[Mesh] OR "Insulin-Like Growth Factor I" OR “Insulin Like Somatomedin Peptide I” OR “Somatomedin C” OR “IGF-I-SmC” OR “IGF-1” OR

Insulin OR "Insulin"[Mesh] OR “c-peptide” OR “c peptide” OR "C-Peptide"[Mesh] OR “Connecting Peptide” OR Phosphorus OR "Phosphorus"[Mesh] OR Parathyroid OR PTH OR “Parathyroid Hormone”  OR Parathormone OR Parathyrin

OR “vit d3” OR "1,25-dihydroxy-16,23-diene vitamin D3" OR “1,25DHD Vit D3” OR "25-hydroxy-16,23-diene vitamin D3" OR “25H-16,23D-Vit D3” OR “25-hydroxy-16,23E-diene vitamin D3” OR "Cholecalciferol"[Mesh] OR Calciol OR “Vitamin D 3” OR “Vitamin D3” OR Cholecalciferols OR “Biochemical markers” OR "Biomarkers"[Mesh] OR Biomarkers OR Marker* OR Glucose OR "Glucose"[Mesh] OR Dextrose OR “Anhydrous Dextrose” OR “L-Glucose” OR “L Glucose” )

**Results: 2111**

**Scopus:**

TITLE-ABS-KEY(fast*) AND (Ramadan OR Ramedan OR Ramezan OR ramazan OR Ramdan OR Ramzan OR religious OR Islamic OR intermittent ) AND ( Hormone OR Hormones OR Prolactin OR Mammotropin OR PRL OR “SRIH-14” OR “Somatostatin-14” OR [Somatostatin](http://www.ncbi.nlm.nih.gov/mesh/68013004) OR Pituitary OR HypophyS* OR (Infundibular AND (Stalk* OR  Stem)) OR Infundibulum* OR Corticoliberin OR CRF-41 CRF OR (((Corticotropin OR ACTH ) AND Releasing) AND (Factor OR Hormone)) OR CRH OR ([Gonadotropin-Releasing AND Hormone](http://www.ncbi.nlm.nih.gov/mesh/68007987)) OR GnRH OR LFRH OR Gn-RH OR LHFSHRH OR LHRH OR LH-RH OR ([Growth AND Hormone-Releasing AND Hormone](http://www.ncbi.nlm.nih.gov/mesh/68013007)) OR GHRH OR “GRF 1-44” OR Somatocrinin OR Somatoliberin OR “GHRH 1-44” OR “hpGRF 44” OR (“[Thyrotropin-Releasing” AND Hormone](http://www.ncbi.nlm.nih.gov/mesh/68013973)) OR TRH OR “Stimu-TSH” OR “Stimu TSH” OR “StimuTSH” OR [Pro-Opiomelanocortin](http://www.ncbi.nlm.nih.gov/mesh/68011333) OR “Pro Opiomelanocortin” OR Proopiomelanocortin OR “Pro-ACTH-Endorphin” OR “Pro ACTH Endorphin” OR “Pro-Opio-Melanocortin” OR “Pro Opio Melanocortin” OR “Pro-Opiocortin” OR “Pro Opiocortin” OR “Proopiocortin” OR “ ACTH-Endorphin Precursor” OR “ACTH Endorphin Precursor” OR “Endorphin-ACTH Precursor” OR “Endorphin ACTH Precursor” OR Preproopiomelanocortin OR “Pre-pro-opiocortin” OR “Pre pro opiocortin” OR “Pre-POMC” OR “Pre POMC” OR Opiocortin OR POMC OR [Gonadotropin*](http://www.ncbi.nlm.nih.gov/mesh/68006065) OR “ Follicle-Stimulating Hormone” OR “ [Follicle Stimulating Hormone](http://www.ncbi.nlm.nih.gov/mesh/68005640)” OR FSH OR [Luteinizing](http://www.ncbi.nlm.nih.gov/mesh/68007986)  OR LH OR “ICSH Interstitial Cell Stimulating Hormone” OR “[Growth Hormone](http://www.ncbi.nlm.nih.gov/mesh/68013006)” OR “[Growth Hormone](http://www.ncbi.nlm.nih.gov/mesh/68013006)S” OR Somatotropin* OR GH OR (Adrenocorticotropic AND Hormone) OR Corticotrophin OR Corticotropin OR “1-39 ACTH” OR Adrenocorticotropin OR ACTH OR “1-24-Corticotropin” OR “1-24-ACTH” OR TSH OR Adrenal OR [Gonad](http://www.ncbi.nlm.nih.gov/mesh/68006066) OR [Gonad](http://www.ncbi.nlm.nih.gov/mesh/68006066)s OR [Thyroid* OR](http://www.ncbi.nlm.nih.gov/mesh/68013961) [Endocrin* OR "Endocrinology" OR](http://www.ncbi.nlm.nih.gov/mesh/68004703)  Ovaries OR [Ovary](http://www.ncbi.nlm.nih.gov/mesh/68010053) OR [Ovarian](http://www.ncbi.nlm.nih.gov/mesh/68006080) OR [Testis](http://www.ncbi.nlm.nih.gov/mesh/68013737) OR Testicles OR Testicle OR Testes OR [Leydig](http://www.ncbi.nlm.nih.gov/mesh/68007985) OR (Testicular AND Interstitial AND Cells) OR [Diiodothyronines](http://www.ncbi.nlm.nih.gov/mesh/68004104) OR [Triiodothyronine](http://www.ncbi.nlm.nih.gov/mesh/68014284) OR Liothyronine OR T3 OR T4 OR Tetraiodothyronine OR [Thyroxine](http://www.ncbi.nlm.nih.gov/mesh/68013974) OR “T3 resin uptake “ OR t3ru OR “T 3 resin uptake” OR “t3 ru” OR “Free t4” OR “Freet4” OR “ Thyroid Function Test” OR “Freet3” OR “Total T4 ” OR “TT4” OR “Total T3” OR “TT3” OR Progesterone OR “[alpha-Dihydroprogesterone](http://www.ncbi.nlm.nih.gov/mesh/68004092)” OR [Hydroxyprogesterone*](http://www.ncbi.nlm.nih.gov/mesh/68006908) OR [Pregnanediol](http://www.ncbi.nlm.nih.gov/mesh/68011276) OR [Estradiol](http://www.ncbi.nlm.nih.gov/mesh/68004958)* OR Oestradiol OR Estrogen* OR Testosterone OR "Adrenal Cortex Corticosteroids” OR Corticoids OR “[17-Ketosteroid](http://www.ncbi.nlm.nih.gov/mesh/68015068)” OR “17 Ketosteroids” OR “17-Oxosteroids” OR “17 Oxosteroids” OR [Androstenedione](http://www.ncbi.nlm.nih.gov/mesh/68000735) OR “delta-4-Androstenedione” OR (“4-Androstene-3” AND “17-dione”) OR “5 alpha-Androstan-3 alpha-ol-17-one” OR “3-alpha-Hydroxy-5-alpha-Androstan-17-One” OR “3 alpha Hydroxy 5 alpha Androstan 17 One” OR [Androsterone](http://www.ncbi.nlm.nih.gov/mesh/68000738) OR Epiandrosterone OR [Dehydroepiandrosterone](http://www.ncbi.nlm.nih.gov/mesh/68003687) OR Dehydroisoandrosterone OR DHEA OR Androstenolone OR “5-Androsten-3-ol-17-one” OR “5 Androsten 3 ol 17 one” OR [Estrone](http://www.ncbi.nlm.nih.gov/mesh/68004970) OR Folliculin OR [corticoids](http://www.ncbi.nlm.nih.gov/mesh/68005938) OR Glucocorticoid OR [Hydroxycorticosteroid*](http://www.ncbi.nlm.nih.gov/mesh/68006889) OR [Desoxycorticosterone](http://www.ncbi.nlm.nih.gov/mesh/68003900) OR Desoxycortone OR Cortexone OR Deoxycorticosterone OR “11-Decorticosterone” OR “21-Hydroxyprogesterone” OR “21 Hydroxyprogesterone” OR [Pregnenolone](http://www.ncbi.nlm.nih.gov/mesh/68011284) OR “5-Pregnen-3-beta-ol-20-one”

OR (Blood AND (Sugar OR Glucose)) OR Proglucagon OR “HG Factor” OR “Hyperglycemic Glycogenolytic Factor” OR Glucagon OR Glukagon OR [Calcium](https://www.ncbi.nlm.nih.gov/mesh/68002118) OR “Factor IV”

OR "Insulin-Like Growth Factor I" OR “Insulin Like Somatomedin Peptide I” OR “Somatomedin C” OR “IGF-I-SmC” OR “IGF-1” OR

Insulin OR “c-peptide” OR “c peptide” OR “Connecting Peptide” OR Phosphorus OR Parathyroid OR PTH OR “Parathyroid Hormone”  OR Parathormone OR Parathyrin

OR “vit d3” OR "1,25-dihydroxy-16,23-diene vitamin D3" OR “1,25DHD Vit D3” OR "25-hydroxy-16,23-diene vitamin D3" OR “25H-16,23D-Vit D3” OR “25-hydroxy-16,23E-diene vitamin D3” OR Calciol OR “Vitamin D 3” OR “Vitamin D3” OR Cholecalciferols OR “Biochemical markers” OR Biomarkers OR Marker* OR Glucose OR Dextrose OR “Anhydrous Dextrose” OR “L-Glucose” OR “L Glucose”)

**Results: 3954**

**Web Of Science:**

TS=((fast*) AND (Ramadan OR Ramedan OR Ramezan OR ramazan OR Ramdan OR Ramzan OR religious OR Islamic OR intermittent ) AND ( Hormone OR Hormones OR Prolactin OR Mammotropin OR PRL OR “SRIH-14” OR “Somatostatin-14” OR [Somatostatin](http://www.ncbi.nlm.nih.gov/mesh/68013004) OR Pituitary OR HypophyS* OR (Infundibular AND (Stalk* OR  Stem)) OR Infundibulum* OR Corticoliberin OR CRF-41 CRF OR (((Corticotropin OR ACTH ) AND Releasing) AND (Factor OR Hormone)) OR CRH OR ([Gonadotropin-Releasing AND Hormone](http://www.ncbi.nlm.nih.gov/mesh/68007987)) OR GnRH OR LFRH OR Gn-RH OR LHFSHRH OR LHRH OR LH-RH OR ([Growth AND Hormone-Releasing AND Hormone](http://www.ncbi.nlm.nih.gov/mesh/68013007)) OR GHRH OR “GRF 1-44” OR Somatocrinin OR Somatoliberin OR “GHRH 1-44” OR “hpGRF 44” OR (“[Thyrotropin-Releasing” AND Hormone](http://www.ncbi.nlm.nih.gov/mesh/68013973)) OR TRH OR “Stimu-TSH” OR “Stimu TSH” OR “StimuTSH” OR [Pro-Opiomelanocortin](http://www.ncbi.nlm.nih.gov/mesh/68011333) OR “Pro Opiomelanocortin” OR Proopiomelanocortin OR “Pro-ACTH-Endorphin” OR “Pro ACTH Endorphin” OR “Pro-Opio-Melanocortin” OR “Pro Opio Melanocortin” OR “Pro-Opiocortin” OR “Pro Opiocortin” OR “Proopiocortin” OR “ ACTH-Endorphin Precursor” OR “ACTH Endorphin Precursor” OR “Endorphin-ACTH Precursor” OR “Endorphin ACTH Precursor” OR Preproopiomelanocortin OR “Pre-pro-opiocortin” OR “Pre pro opiocortin” OR “Pre-POMC” OR “Pre POMC” OR Opiocortin OR POMC OR [Gonadotropin*](http://www.ncbi.nlm.nih.gov/mesh/68006065) OR “ Follicle-Stimulating Hormone” OR “ [Follicle Stimulating Hormone](http://www.ncbi.nlm.nih.gov/mesh/68005640)” OR FSH OR [Luteinizing](http://www.ncbi.nlm.nih.gov/mesh/68007986)  OR LH OR “ICSH Interstitial Cell Stimulating Hormone” OR “[Growth Hormone](http://www.ncbi.nlm.nih.gov/mesh/68013006)” OR “[Growth Hormone](http://www.ncbi.nlm.nih.gov/mesh/68013006)S” OR Somatotropin* OR GH OR (Adrenocorticotropic AND Hormone) OR Corticotrophin OR Corticotropin OR “1-39 ACTH” OR Adrenocorticotropin OR ACTH OR “1-24-Corticotropin” OR “1-24-ACTH” OR TSH OR Adrenal OR [Gonad](http://www.ncbi.nlm.nih.gov/mesh/68006066) OR [Gonad](http://www.ncbi.nlm.nih.gov/mesh/68006066)s OR [Thyroid* OR](http://www.ncbi.nlm.nih.gov/mesh/68013961) [Endocrin* OR "Endocrinology" OR](http://www.ncbi.nlm.nih.gov/mesh/68004703)  Ovaries OR [Ovary](http://www.ncbi.nlm.nih.gov/mesh/68010053) OR [Ovarian](http://www.ncbi.nlm.nih.gov/mesh/68006080) OR [Testis](http://www.ncbi.nlm.nih.gov/mesh/68013737) OR Testicles OR Testicle OR Testes OR [Leydig](http://www.ncbi.nlm.nih.gov/mesh/68007985) OR (Testicular AND Interstitial AND Cells) OR [Diiodothyronines](http://www.ncbi.nlm.nih.gov/mesh/68004104) OR [Triiodothyronine](http://www.ncbi.nlm.nih.gov/mesh/68014284) OR Liothyronine OR T3 OR T4 OR Tetraiodothyronine OR [Thyroxine](http://www.ncbi.nlm.nih.gov/mesh/68013974) OR “T3 resin uptake “ OR t3ru OR “T 3 resin uptake” OR “t3 ru” OR “Free t4” OR “Freet4” OR “ Thyroid Function Test” OR “Freet3” OR “Total T4 ” OR “TT4” OR “Total T3” OR “TT3” OR Progesterone OR “[alpha-Dihydroprogesterone](http://www.ncbi.nlm.nih.gov/mesh/68004092)” OR [Hydroxyprogesterone*](http://www.ncbi.nlm.nih.gov/mesh/68006908) OR [Pregnanediol](http://www.ncbi.nlm.nih.gov/mesh/68011276) OR [Estradiol](http://www.ncbi.nlm.nih.gov/mesh/68004958)* OR Oestradiol OR Estrogen* OR Testosterone OR "Adrenal Cortex Corticosteroids” OR Corticoids OR “[17-Ketosteroid](http://www.ncbi.nlm.nih.gov/mesh/68015068)” OR “17 Ketosteroids” OR “17-Oxosteroids” OR “17 Oxosteroids” OR [Androstenedione](http://www.ncbi.nlm.nih.gov/mesh/68000735) OR “delta-4-Androstenedione” OR (“4-Androstene-3” AND “17-dione”) OR “5 alpha-Androstan-3 alpha-ol-17-one” OR “3-alpha-Hydroxy-5-alpha-Androstan-17-One” OR “3 alpha Hydroxy 5 alpha Androstan 17 One” OR [Androsterone](http://www.ncbi.nlm.nih.gov/mesh/68000738) OR Epiandrosterone OR [Dehydroepiandrosterone](http://www.ncbi.nlm.nih.gov/mesh/68003687) OR Dehydroisoandrosterone OR DHEA OR Androstenolone OR “5-Androsten-3-ol-17-one” OR “5 Androsten 3 ol 17 one” OR [Estrone](http://www.ncbi.nlm.nih.gov/mesh/68004970) OR Folliculin OR [corticoids](http://www.ncbi.nlm.nih.gov/mesh/68005938) OR Glucocorticoid OR [Hydroxycorticosteroid*](http://www.ncbi.nlm.nih.gov/mesh/68006889) OR [Desoxycorticosterone](http://www.ncbi.nlm.nih.gov/mesh/68003900) OR Desoxycortone OR Cortexone OR Deoxycorticosterone OR “11-Decorticosterone” OR “21-Hydroxyprogesterone” OR “21 Hydroxyprogesterone” OR [Pregnenolone](http://www.ncbi.nlm.nih.gov/mesh/68011284) OR “5-Pregnen-3-beta-ol-20-one”

OR (Blood AND (Sugar OR Glucose)) OR Proglucagon OR “HG Factor” OR “Hyperglycemic Glycogenolytic Factor” OR Glucagon OR Glukagon OR [Calcium](https://www.ncbi.nlm.nih.gov/mesh/68002118) OR “Factor IV”

OR "Insulin-Like Growth Factor I" OR “Insulin Like Somatomedin Peptide I” OR “Somatomedin C” OR “IGF-I-SmC” OR “IGF-1” OR

Insulin OR “c-peptide” OR “c peptide” OR “Connecting Peptide” OR Phosphorus OR Parathyroid OR PTH OR “Parathyroid Hormone”  OR Parathormone OR Parathyrin

OR “vit d3” OR "1,25-dihydroxy-16,23-diene vitamin D3" OR “1,25DHD Vit D3” OR "25-hydroxy-16,23-diene vitamin D3" OR “25H-16,23D-Vit D3” OR “25-hydroxy-16,23E-diene vitamin D3” OR Calciol OR “Vitamin D 3” OR “Vitamin D3” OR Cholecalciferols OR “Biochemical markers” OR Biomarkers OR Marker* OR Glucose OR Dextrose OR “Anhydrous Dextrose” OR “L-Glucose” OR “L Glucose”))

**Results: 2049**

**Embase:**

(('diet restriction'/exp OR fast***:ab,ti**) AND (Ramadan OR Ramedan OR Ramezan OR ramazan OR Ramdan OR Ramzan OR religious OR Islamic OR intermittent ) AND ( Hormone OR Hormones OR Prolactin OR Mammotropin OR PRL OR “SRIH-14” OR “Somatostatin-14” OR [Somatostatin](http://www.ncbi.nlm.nih.gov/mesh/68013004) OR Pituitary OR HypophyS* OR (Infundibular AND (Stalk* OR  Stem)) OR Infundibulum* OR Corticoliberin OR CRF-41 CRF OR (((Corticotropin OR ACTH ) AND Releasing) AND (Factor OR Hormone)) OR CRH OR ([Gonadotropin-Releasing AND Hormone](http://www.ncbi.nlm.nih.gov/mesh/68007987)) OR GnRH OR LFRH OR Gn-RH OR LHFSHRH OR LHRH OR LH-RH OR ([Growth AND Hormone-Releasing AND Hormone](http://www.ncbi.nlm.nih.gov/mesh/68013007)) OR GHRH OR “GRF 1-44” OR Somatocrinin OR Somatoliberin OR “GHRH 1-44” OR “hpGRF 44” OR (“[Thyrotropin-Releasing” AND Hormone](http://www.ncbi.nlm.nih.gov/mesh/68013973)) OR TRH OR “Stimu-TSH” OR “Stimu TSH” OR “StimuTSH” OR [Pro-Opiomelanocortin](http://www.ncbi.nlm.nih.gov/mesh/68011333) OR “Pro Opiomelanocortin” OR Proopiomelanocortin OR “Pro-ACTH-Endorphin” OR “Pro ACTH Endorphin” OR “Pro-Opio-Melanocortin” OR “Pro Opio Melanocortin” OR “Pro-Opiocortin” OR “Pro Opiocortin” OR “Proopiocortin” OR “ ACTH-Endorphin Precursor” OR “ACTH Endorphin Precursor” OR “Endorphin-ACTH Precursor” OR “Endorphin ACTH Precursor” OR Preproopiomelanocortin OR “Pre-pro-opiocortin” OR “Pre pro opiocortin” OR “Pre-POMC” OR “Pre POMC” OR Opiocortin OR POMC OR [Gonadotropin*](http://www.ncbi.nlm.nih.gov/mesh/68006065) OR “ Follicle-Stimulating Hormone” OR “ [Follicle Stimulating Hormone](http://www.ncbi.nlm.nih.gov/mesh/68005640)” OR FSH OR [Luteinizing](http://www.ncbi.nlm.nih.gov/mesh/68007986)  OR LH OR “ICSH Interstitial Cell Stimulating Hormone” OR “[Growth Hormone](http://www.ncbi.nlm.nih.gov/mesh/68013006)” OR “[Growth Hormone](http://www.ncbi.nlm.nih.gov/mesh/68013006)S” OR Somatotropin* OR GH OR (Adrenocorticotropic AND Hormone) OR Corticotrophin OR Corticotropin OR “1-39 ACTH” OR Adrenocorticotropin OR ACTH OR “1-24-Corticotropin” OR “1-24-ACTH” OR TSH OR Adrenal OR [Gonad](http://www.ncbi.nlm.nih.gov/mesh/68006066) OR [Gonad](http://www.ncbi.nlm.nih.gov/mesh/68006066)s OR [Thyroid* OR](http://www.ncbi.nlm.nih.gov/mesh/68013961) [Endocrin* OR "Endocrinology" OR](http://www.ncbi.nlm.nih.gov/mesh/68004703)  Ovaries OR [Ovary](http://www.ncbi.nlm.nih.gov/mesh/68010053) OR [Ovarian](http://www.ncbi.nlm.nih.gov/mesh/68006080) OR [Testis](http://www.ncbi.nlm.nih.gov/mesh/68013737) OR Testicles OR Testicle OR Testes OR [Leydig](http://www.ncbi.nlm.nih.gov/mesh/68007985) OR (Testicular AND Interstitial AND Cells) OR [Diiodothyronines](http://www.ncbi.nlm.nih.gov/mesh/68004104) OR [Triiodothyronine](http://www.ncbi.nlm.nih.gov/mesh/68014284) OR Liothyronine OR T3 OR T4 OR Tetraiodothyronine OR [Thyroxine](http://www.ncbi.nlm.nih.gov/mesh/68013974) OR “T3 resin uptake “ OR t3ru OR “T 3 resin uptake” OR “t3 ru” OR “Free t4” OR “Freet4” OR “ Thyroid Function Test” OR “Freet3” OR “Total T4 ” OR “TT4” OR “Total T3” OR “TT3” OR Progesterone OR “[alpha-Dihydroprogesterone](http://www.ncbi.nlm.nih.gov/mesh/68004092)” OR [Hydroxyprogesterone*](http://www.ncbi.nlm.nih.gov/mesh/68006908) OR [Pregnanediol](http://www.ncbi.nlm.nih.gov/mesh/68011276) OR [Estradiol](http://www.ncbi.nlm.nih.gov/mesh/68004958)* OR Oestradiol OR Estrogen* OR Testosterone OR "Adrenal Cortex Corticosteroids” OR Corticoids OR “[17-Ketosteroid](http://www.ncbi.nlm.nih.gov/mesh/68015068)” OR “17 Ketosteroids” OR “17-Oxosteroids” OR “17 Oxosteroids” OR [Androstenedione](http://www.ncbi.nlm.nih.gov/mesh/68000735) OR “delta-4-Androstenedione” OR (“4-Androstene-3” AND “17-dione”) OR “5 alpha-Androstan-3 alpha-ol-17-one” OR “3-alpha-Hydroxy-5-alpha-Androstan-17-One” OR “3 alpha Hydroxy 5 alpha Androstan 17 One” OR [Androsterone](http://www.ncbi.nlm.nih.gov/mesh/68000738) OR Epiandrosterone OR [Dehydroepiandrosterone](http://www.ncbi.nlm.nih.gov/mesh/68003687) OR Dehydroisoandrosterone OR DHEA OR Androstenolone OR “5-Androsten-3-ol-17-one” OR “5 Androsten 3 ol 17 one” OR [Estrone](http://www.ncbi.nlm.nih.gov/mesh/68004970) OR Folliculin OR [corticoids](http://www.ncbi.nlm.nih.gov/mesh/68005938) OR Glucocorticoid OR [Hydroxycorticosteroid*](http://www.ncbi.nlm.nih.gov/mesh/68006889) OR [Desoxycorticosterone](http://www.ncbi.nlm.nih.gov/mesh/68003900) OR Desoxycortone OR Cortexone OR Deoxycorticosterone OR “11-Decorticosterone” OR “21-Hydroxyprogesterone” OR “21 Hydroxyprogesterone” OR [Pregnenolone](http://www.ncbi.nlm.nih.gov/mesh/68011284) OR “5-Pregnen-3-beta-ol-20-one”

OR (Blood AND (Sugar OR Glucose)) OR Proglucagon OR “HG Factor” OR “Hyperglycemic Glycogenolytic Factor” OR Glucagon OR Glukagon OR [Calcium](https://www.ncbi.nlm.nih.gov/mesh/68002118) OR “Factor IV” OR "Insulin-Like Growth Factor I" OR “Insulin Like Somatomedin Peptide I” OR “Somatomedin C” OR “IGF-I-SmC” OR “IGF-1” OR

Insulin OR “c-peptide” OR “c peptide” OR “Connecting Peptide” OR Phosphorus OR Parathyroid OR PTH OR “Parathyroid Hormone”  OR Parathormone OR Parathyrin

OR “vit d3” OR "1,25-dihydroxy-16,23-diene vitamin D3" OR “1,25DHD Vit D3” OR "25-hydroxy-16,23-diene vitamin D3" OR “25H-16,23D-Vit D3” OR “25-hydroxy-16,23E-diene vitamin D3” OR Calciol OR “Vitamin D 3” OR “Vitamin D3” OR Cholecalciferols OR “Biochemical markers” OR Biomarkers OR Marker* OR Glucose OR Dextrose OR “Anhydrous Dextrose” OR “L-Glucose” OR “L Glucose” OR 'hormone'/exp OR 'prolactin'/exp OR 'somatostatin'/exp OR 'corticotropin releasing factor'/exp OR 'gonadorelin'/exp OR 'growth hormone releasing factor'/exp OR 'proopiomelanocortin'/exp OR 'follitropin'/exp OR 'luteinizing hormone'/exp OR 'growth hormone'/exp OR 'corticotropin'/exp OR 'adrenal gland'/exp OR 'thyroid gland'/exp OR 'gonadotropin'/exp OR 'endocrinology'/exp OR 'ovary'/exp OR 'testis'/exp OR 'testis'/exp OR 'liothyronine'/exp OR 'thyroxine'/exp OR 'thyroid function test'/exp OR 'progesterone'/exp OR '20alpha dihydroprogesterone'/exp OR '5alpha pregnane 3,20 dione'/exp OR 'progesterone derivative'/exp OR 'pregnanediol'/exp OR 'estradiol'/exp OR 'estrogen'/exp OR '1 testosterone'/exp OR 'corticosteroid'/exp OR '17 oxosteroid'/exp OR 'androstenedione'/exp OR 'prasterone'/exp OR 'glucocorticoid'/exp OR 'hydroxycorticosteroid'/exp OR 'deoxycorticosterone'/exp OR 'pregnenolone'/exp OR 'glucose blood level'/exp OR 'glucagon'/exp OR 'calcium'/exp OR 'somatomedin C'/exp OR 'insulin'/exp OR 'C peptide'/exp OR 'phosphorus'/exp OR 'colecalciferol'/exp OR 'biological marker'/exp OR 'biological marker'/exp))

**Results: 4447**

**COCHRANE LIBRARY**

ID Search Hits

#1 (fast*):ti,ab,kw (Word variations have been searched)

#2 MeSH descriptor: [Fasting] explode all trees

#3 ((Ramadan or Ramedan or Ramezan or ramazan or Ramdan or Ramzan or religious or Islamic or intermittent)):ti,ab,kw (Word variations have been searched)

#4 ((Hormone or Hormones or Prolactin or Mammotropin or PRL or "SRIH-14" or "Somatostatin-14" or Somatostatin or Pituitary or HypophyS* or (Infundibular and (Stalk* or Stem)) or Infundibulum* or Corticoliberin or CRF-41 CRF or (((Corticotropin or ACTH) and Releasing) and (Factor or Hormone)) or CRH or (Gonadotropin-Releasing and Hormone) or GnRH or LFRH or Gn-RH or LHFSHRH or LHRH or LH-RH or (Growth and Hormone-Releasing and Hormone) or GHRH or "GRF 1-44" or Somatocrinin or Somatoliberin or "GHRH 1-44" or "hpGRF 44" or ("Thyrotropin-Releasing" and Hormone) or TRH or "Stimu-TSH" or "Stimu TSH" or "StimuTSH" or Pro-Opiomelanocortin or "Pro Opiomelanocortin" or Proopiomelanocortin or "Pro-ACTH-Endorphin" or "Pro ACTH Endorphin" or "Pro-Opio-Melanocortin" or "Pro Opio Melanocortin" or "Pro-Opiocortin" or "Pro Opiocortin" or "Proopiocortin" or " ACTH-Endorphin Precursor" or "ACTH Endorphin Precursor" or "Endorphin-ACTH Precursor" or "Endorphin ACTH Precursor" or Preproopiomelanocortin or "Pre-pro-opiocortin" or "Pre pro opiocortin" or "Pre-POMC" or "Pre POMC" or Opiocortin or POMC or Gonadotropin* or " Follicle-Stimulating Hormone" or " Follicle Stimulating Hormone" or FSH or Luteinizing or LH or "ICSH Interstitial Cell Stimulating Hormone" or "Growth Hormone" or "Growth HormoneS" or Somatotropin* or GH or (Adrenocorticotropic and Hormone) or Corticotrophin or Corticotropin or "1-39 ACTH" or Adrenocorticotropin or ACTH or "1-24-Corticotropin" or "1-24-ACTH" or TSH or Adrenal or Gonad or Gonads or Thyroid* or Endocrin* or "Endocrinology" or Ovaries or Ovary or Ovarian or Testis or Testicles or Testicle or Testes or Leydig or (Testicular and Interstitial and Cells) or Diiodothyronines or Triiodothyronine or Liothyronine or T3 or T4 or Tetraiodothyronine or Thyroxine or "T3 resin uptake " or t3ru or "T 3 resin uptake" or "t3 ru" or "Free t4" or "Freet4" or " Thyroid Function Test" or "Freet3" or "Total T4 " or "TT4" or "Total T3" or "TT3" or Progesterone or "alpha-Dihydroprogesterone" or Hydroxyprogesterone* or Pregnanediol or Estradiol* or Oestradiol or Estrogen* or Testosterone or "Adrenal Cortex Corticosteroids" or Corticoids or "17-Ketosteroid" or "17 Ketosteroids" or "17-Oxosteroids" or "17 Oxosteroids" or Androstenedione or "delta-4-Androstenedione" or ("4-Androstene-3" and "17-dione") or "5 alpha-Androstan-3 alpha-ol-17-one" or "3-alpha-Hydroxy-5-alpha-Androstan-17-One" or "3 alpha Hydroxy 5 alpha Androstan 17 One" or Androsterone or Epiandrosterone or Dehydroepiandrosterone or Dehydroisoandrosterone or DHEA or Androstenolone or "5-Androsten-3-ol-17-one" or "5 Androsten 3 ol 17 one" or Estrone or Folliculin or corticoids or Glucocorticoid or Hydroxycorticosteroid* or Desoxycorticosterone or Desoxycortone or Cortexone or Deoxycorticosterone or "11-Decorticosterone" or "21-Hydroxyprogesterone" or "21 Hydroxyprogesterone" or Pregnenolone or "5-Pregnen-3-beta-ol-20-one" or (Blood and (Sugar or Glucose)) or Proglucagon or "HG Factor" or "Hyperglycemic Glycogenolytic Factor" or Glucagon or Glukagon or Calcium or "Factor IV" or "Insulin-Like Growth Factor I" or "Insulin Like Somatomedin Peptide I" or "Somatomedin C" or "IGF-I-SmC" or "IGF-1" or Insulin or insulins or "c-peptide" or "c peptide" or "Connecting Peptide" or Phosphorus or Parathyroid or PTH or "Parathyroid Hormone" or Parathormone or Parathyrin or "vit d3" or "1,25-dihydroxy-16,23-diene vitamin D3" or "1,25DHD Vit D3" or "25-hydroxy-16,23-diene vitamin D3" or "25H-16,23D-Vit D3" or "25-hydroxy-16,23E-diene vitamin D3" or Calciol or "Vitamin D 3" or "Vitamin D3" or Cholecalciferols or "Biochemical markers" or Biomarkers or Marker* or Glucose or Dextrose or "Anhydrous Dextrose" or "L-Glucose" or "L Glucose")):ti,ab,kw (Word variations have been searched)

#5 MeSH descriptor: [Hormones] explode all trees

#6 MeSH descriptor: [Prolactin] explode all trees

#7 MeSH descriptor: [Somatostatin] explode all trees

#8 MeSH descriptor: [Corticotropin-Releasing Hormone] explode all trees

#9 MeSH descriptor: [Gonadotropin-Releasing Hormone] explode all trees

#10 MeSH descriptor: [Growth Hormone-Releasing Hormone] explode all trees

#11 MeSH descriptor: [Pro-Opiomelanocortin] explode all trees

#12 MeSH descriptor: [Follicle Stimulating Hormone] explode all trees

#13 MeSH descriptor: [Receptors, LHRH] explode all trees

#14 MeSH descriptor: [Growth Hormone] explode all trees

#15 MeSH descriptor: [Adrenocorticotropic Hormone] explode all trees

#16 MeSH descriptor: [Adrenal Glands] explode all trees

#17 MeSH descriptor: [Thyroid Gland] explode all trees

#18 MeSH descriptor: [Gonadotropins, Equine] explode all trees

#19 MeSH descriptor: [Endocrinology] explode all trees

#20 MeSH descriptor: [Ovary] explode all trees

#21 MeSH descriptor: [Testis] explode all trees

#22 MeSH descriptor: [Triiodothyronine] explode all trees

#23 MeSH descriptor: [Thyroxine] explode all trees

#24 MeSH descriptor: [Thyroid Function Tests] explode all trees

#25 MeSH descriptor: [Progesterone] explode all trees

#26 MeSH descriptor: [20-alpha-Dihydroprogesterone] explode all trees

#27 MeSH descriptor: [5-alpha-Dihydroprogesterone] explode all trees

#28 MeSH descriptor: [Hydroxyprogesterones] explode all trees

#29 MeSH descriptor: [Pregnanediol] explode all trees

#30 MeSH descriptor: [Estradiol] explode all trees

#31 MeSH descriptor: [Estrogens] explode all trees

#32 MeSH descriptor: [Testosterone] explode all trees

#33 MeSH descriptor: [Adrenal Cortex Hormones] explode all trees

#34 MeSH descriptor: [17-Ketosteroids] explode all trees

#35 MeSH descriptor: [Androstenedione] explode all trees

#36 MeSH descriptor: [Dehydroepiandrosterone] explode all trees

#37 MeSH descriptor: [Glucocorticoids] explode all trees

#38 MeSH descriptor: [Hydroxycorticosteroids] explode all trees

#39 MeSH descriptor: [Desoxycorticosterone] explode all trees

#40 MeSH descriptor: [Pregnenolone] explode all trees

#41 MeSH descriptor: [Glucagon] explode all trees

#42 MeSH descriptor: [Calcium] explode all trees

#43 MeSH descriptor: [Somatomedins] explode all trees

#44 MeSH descriptor: [Insulin] explode all trees

#45 MeSH descriptor: [Insulins] explode all trees

#46 MeSH descriptor: [C-Peptide] explode all trees

#47 MeSH descriptor: [Phosphorus] explode all trees

#48 MeSH descriptor: [Cholecalciferol] explode all trees

#49 MeSH descriptor: [Biomarkers] explode all trees

#50 MeSH descriptor: [Glucose] explode all trees

#51 #4 or #5 or #6 or #7 or #8 or #9 or #10 or #11 or #12 or #13 or #14 or #15 or #16 or #17 or #18 or #19 or #20 or #21 or #22 or #23 or #24 or #25 or #26 or #27 or #28 or #29 or #30 or #31 or #32 or #33 or #34 or #35 or #36 or #37 or #38 or #39 or #40 or #41 or #42 or #43 or #44 or #45 or #46 or #47 or #48 or #49 or #50

#52 #1 or #2

#53 #3 and #51 and #52

**Results: 512**

**PRISMA**

Additional records identified through references or google scholar
(n = 12)

Records identified through database searching
(n = 13073)

## Included

## Included

## Screening

## Identification

Duplicates removed
(n = 4605)

Records excluded
(n = 8386)

Records screened based on title and abstract
(n = 8480)

## Eligibility

Full-text articles assessed for eligibility
(n = 94)

Full-text articles excluded
(n = 59)

Full-text articles assessed for eligibility
(n = 58 )

Studies included in quantitative synthesis (meta-analysis)
(n = 20 )

Studies included in qualitative synthesis
(n = 57 )

Full-text articles assessed for eligibility
(n = 58 )

Full-text articles assessed for eligibility
(n = 58 )

## Included

Studies included in qualitative synthesis
(n = 35)

## Included

Studies included in quantitative synthesis (meta-analysis)
(n = 28)
