# Supplementary figures and images for: Impact of Ramadan fasting on serum levels of major endocrinology hormonal and biochemical parameters in healthy non-athlete adults: A systematic review and meta-analyses
Source: PLoS One. 2024 May 23;19(5):e0299695. doi: 10.1371/journal.pone.0299695 (PMC11115274; doi:10.1371/journal.pone.0299695)

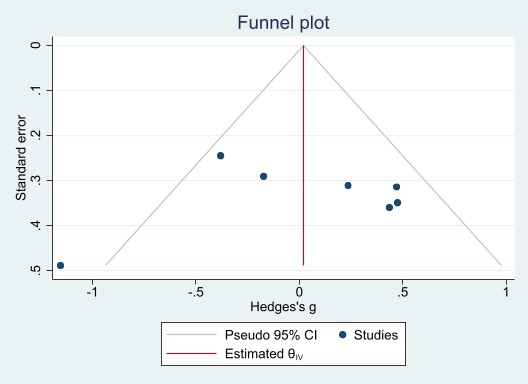

Supplement: S1 Data — (ZIP) [file pone.0299695.s002.zip › Supplements/Funnel Plots/Ca.jpg]

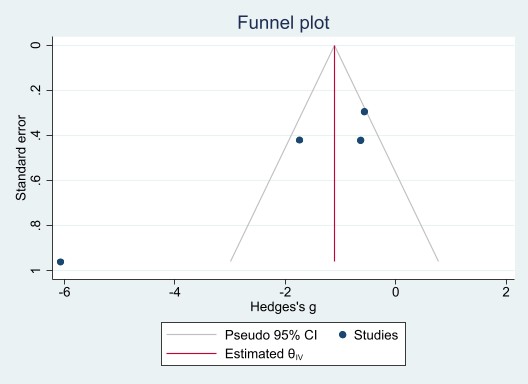

Supplement: S1 Data — (ZIP) [file pone.0299695.s002.zip › Supplements/Funnel Plots/Cortisol.jpg]

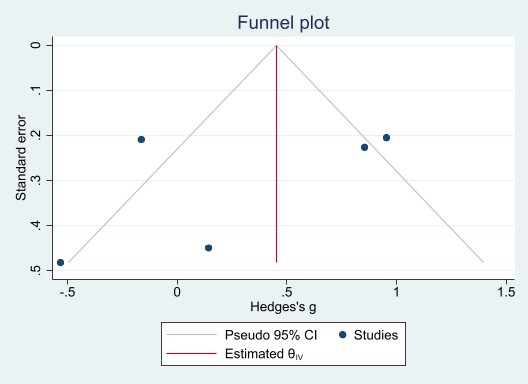

Supplement: S1 Data — (ZIP) [file pone.0299695.s002.zip › Supplements/Funnel Plots/FSH.jpg]

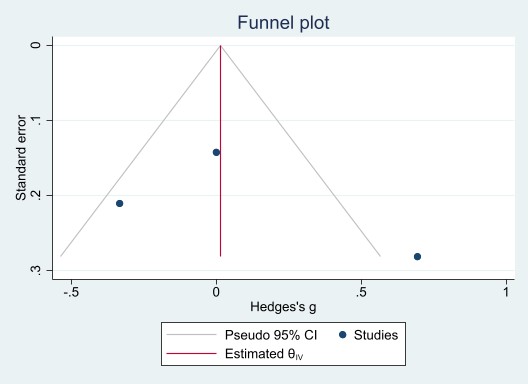

Supplement: S1 Data — (ZIP) [file pone.0299695.s002.zip › Supplements/Funnel Plots/FT3.jpg]

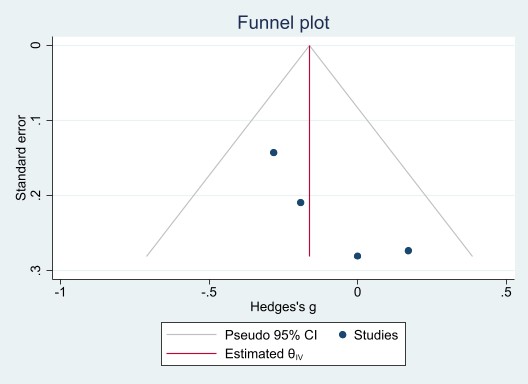

Supplement: S1 Data — (ZIP) [file pone.0299695.s002.zip › Supplements/Funnel Plots/FT4.jpg]

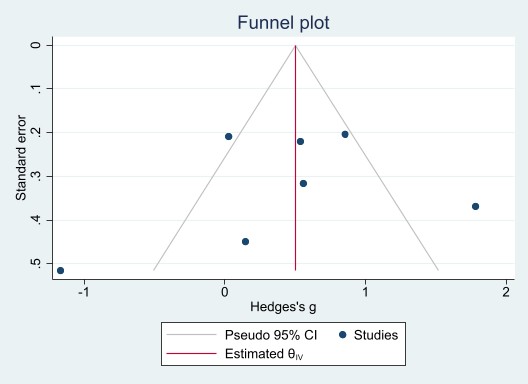

Supplement: S1 Data — (ZIP) [file pone.0299695.s002.zip › Supplements/Funnel Plots/LH.jpg]

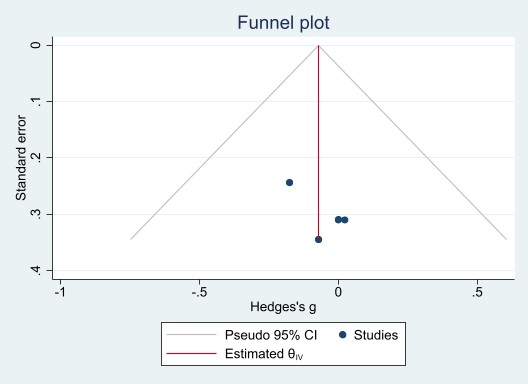

Supplement: S1 Data — (ZIP) [file pone.0299695.s002.zip › Supplements/Funnel Plots/PH.jpg]

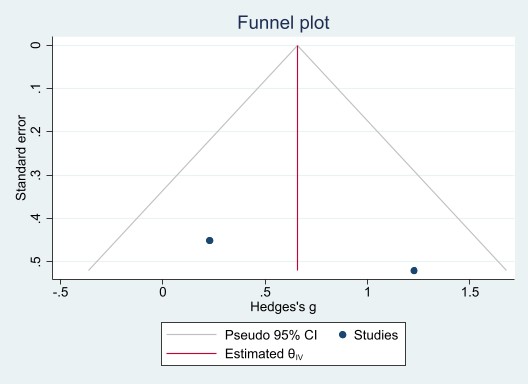

Supplement: S1 Data — (ZIP) [file pone.0299695.s002.zip › Supplements/Funnel Plots/PRL.jpg]

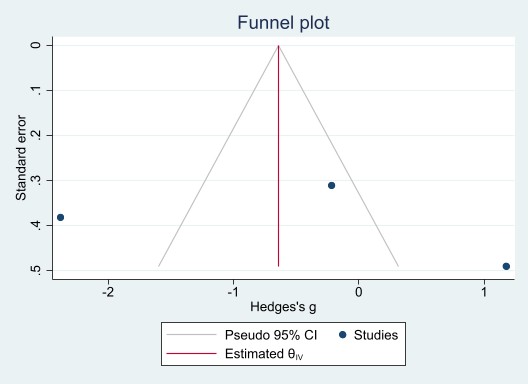

Supplement: S1 Data — (ZIP) [file pone.0299695.s002.zip › Supplements/Funnel Plots/PTH.jpg]

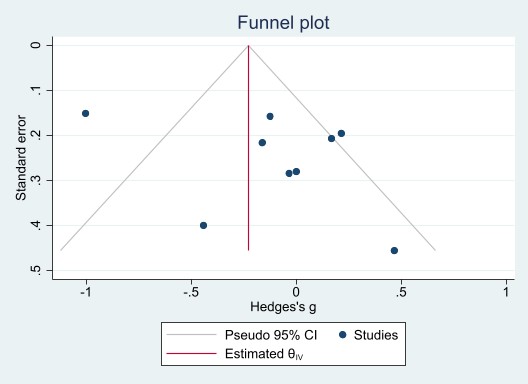

Supplement: S1 Data — (ZIP) [file pone.0299695.s002.zip › Supplements/Funnel Plots/T3.jpg]

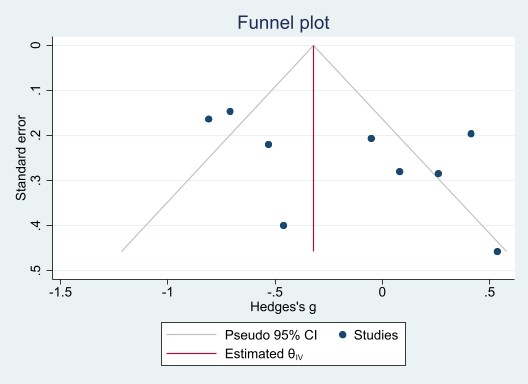

Supplement: S1 Data — (ZIP) [file pone.0299695.s002.zip › Supplements/Funnel Plots/T4.jpg]

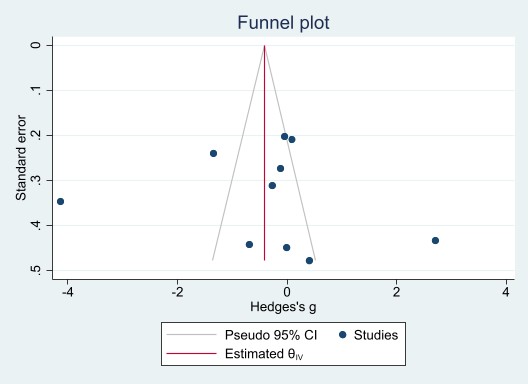

Supplement: S1 Data — (ZIP) [file pone.0299695.s002.zip › Supplements/Funnel Plots/Test.jpg]

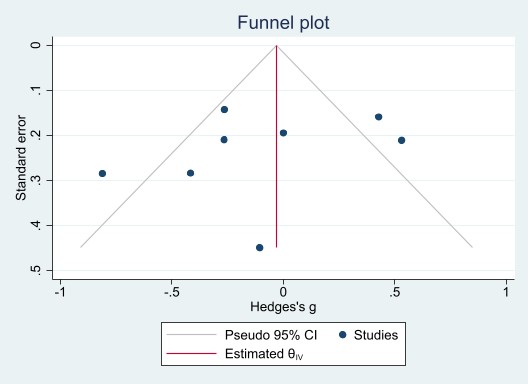

Supplement: S1 Data — (ZIP) [file pone.0299695.s002.zip › Supplements/Funnel Plots/TSH.jpg]

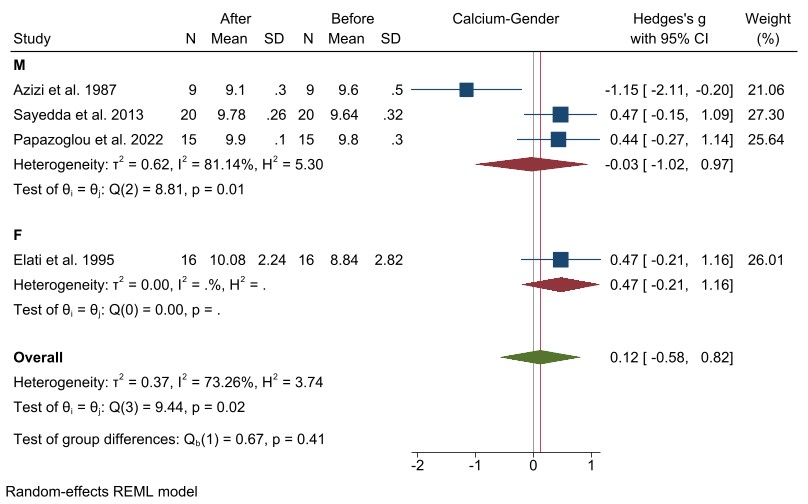

Supplement: S1 Data — (ZIP) [file pone.0299695.s002.zip › Supplements/Gender-Subgroup/Calcium-Gender.jpg]

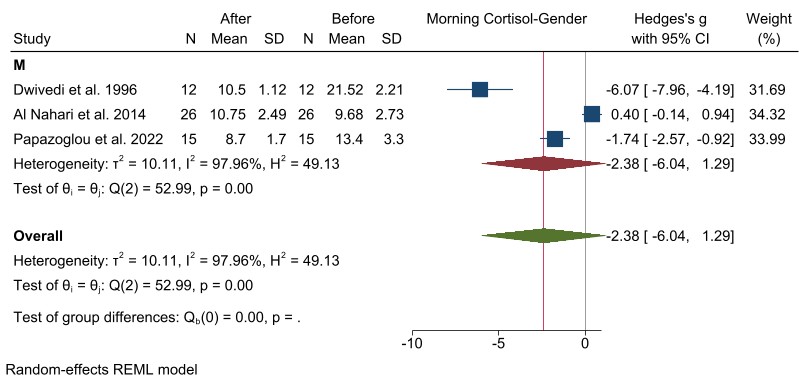

Supplement: S1 Data — (ZIP) [file pone.0299695.s002.zip › Supplements/Gender-Subgroup/Cortisol-Gender.jpg]

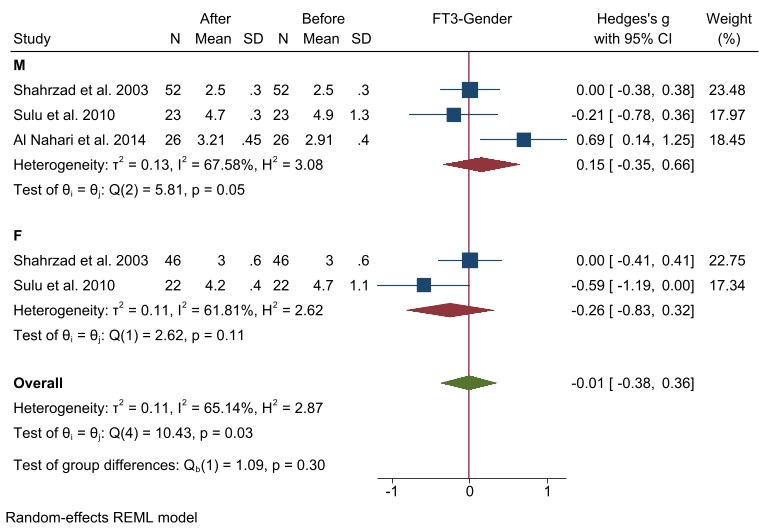

Supplement: S1 Data — (ZIP) [file pone.0299695.s002.zip › Supplements/Gender-Subgroup/FT3-Gender.jpg]

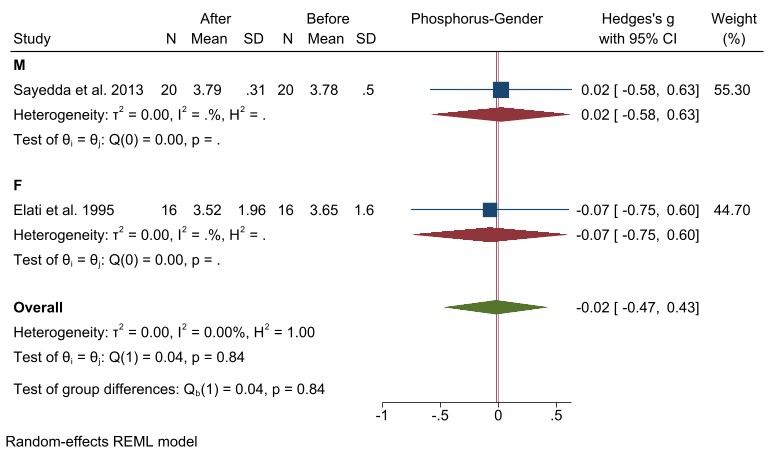

Supplement: S1 Data — (ZIP) [file pone.0299695.s002.zip › Supplements/Gender-Subgroup/Ph-Gender.jpg]

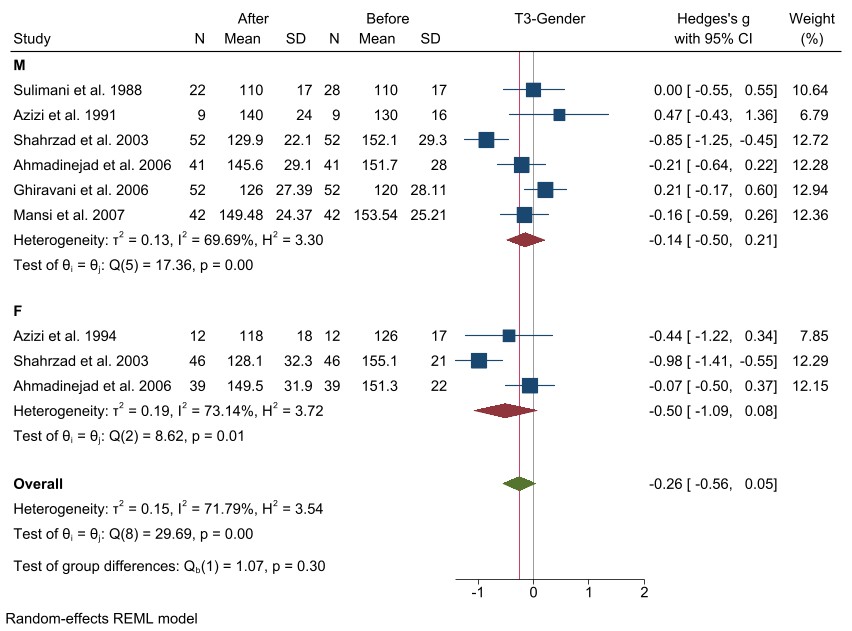

Supplement: S1 Data — (ZIP) [file pone.0299695.s002.zip › Supplements/Gender-Subgroup/T3-Gender.jpg]

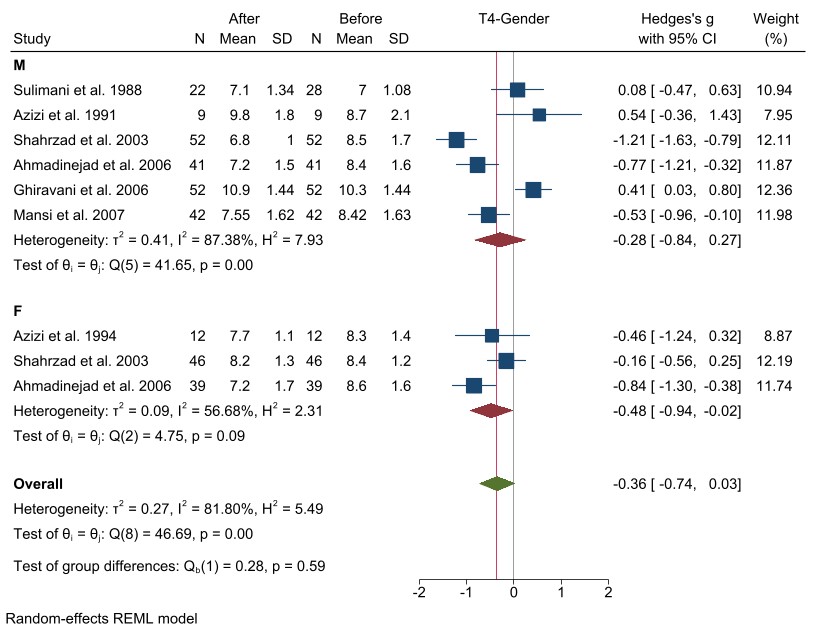

Supplement: S1 Data — (ZIP) [file pone.0299695.s002.zip › Supplements/Gender-Subgroup/T4-Gender.jpg]

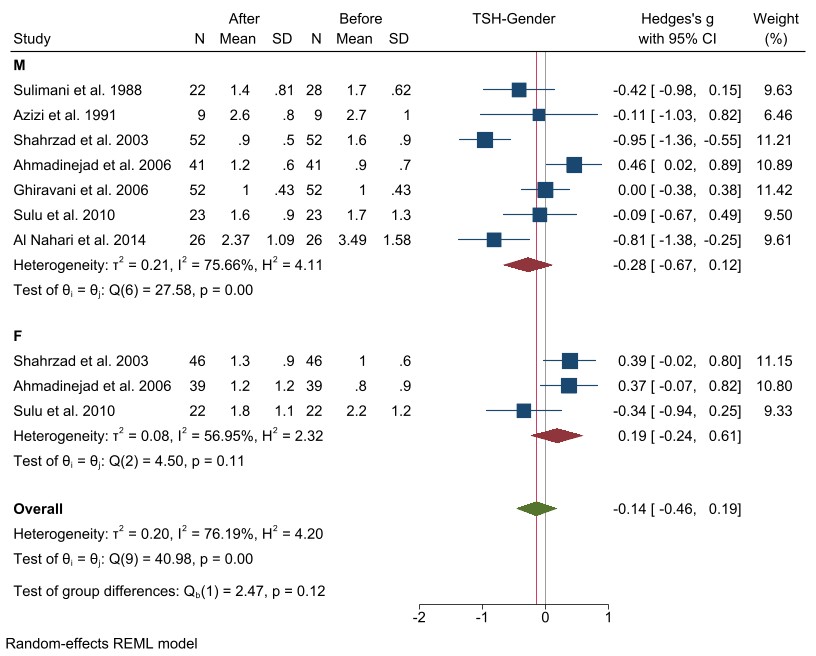

Supplement: S1 Data — (ZIP) [file pone.0299695.s002.zip › Supplements/Gender-Subgroup/tshgender.jpg]
